# Web-Based Interfaces for Virtual *C. elegans* Neuron Model Definition, Network Configuration, Behavioral Experiment Definition and Experiment Results Visualization

**DOI:** 10.3389/fninf.2018.00080

**Published:** 2018-11-13

**Authors:** Gorka Epelde, Fearghal Morgan, Andoni Mujika, Frank Callaly, Peter Leškovský, Brian McGinley, Roberto Álvarez, Axel Blau, Finn Krewer

**Affiliations:** ^1^Vicomtech, San Sebastián, Spain; ^2^IIS Biodonostia, San Sebastián, Spain; ^3^Bio-Inspired Electronics and Reconfigurable Computing (BIRC) Research Group, Electrical & Electronic Engineering Department, College of Engineering and Informatics, National University of Ireland Galway, Galway, Ireland; ^4^Department of Neuroscience and Brain Technologies, Fondazione Istituto Italiano di Tecnologia, Genoa, Italy

**Keywords:** *C. elegans* experiment definition and results visualization, *in silico* simulation, brain-inspired computation, biological neural networks, behavioral experiment input encoding, NeuroML, low entropy model specification (LEMS), neuronal activity visualization

## Abstract

The *Si elegans* platform targets the complete virtualization of the nematode *Caenorhabditis elegans*, and its environment. This paper presents a suite of unified web-based Graphical User Interfaces (GUIs) as the main user interaction point, and discusses their underlying technologies and methods. The user-friendly features of this tool suite enable users to graphically create neuron and network models, and behavioral experiments, without requiring knowledge of domain-specific computer-science tools. The framework furthermore allows the graphical visualization of all simulation results using a worm locomotion and neural activity viewer. Models, experiment definitions and results can be exported in a machine-readable format, thereby facilitating reproducible and cross-platform execution of *in silico C. elegans* experiments in other simulation environments. This is made possible by a novel XML-based behavioral experiment definition encoding format, a NeuroML XML-based model generation and network configuration description language, and their associated GUIs. User survey data confirms the platform usability and functionality, and provides insights into future directions for web-based simulation GUIs of *C. elegans* and other living organisms. The tool suite is available online to the scientific community and its source code has been made available.

## 1. Introduction

*Caenorhabditis elegans* (*C. elegans*), a roundworm, 1 mm long and 80 μm in diameter, is one of the best characterized biological organisms (Altun and Hall, 2002). The adult hermaphrodite comprises 959 cells, including 95 body wall muscle cells and 302 neurons. Despite the relatively small number of cells, the *C. elegans* nervous system generates a rich variety of behavioral patterns in response to internal and external stimuli. Behaviors include mating, foraging, response to sensory stimuli and social interaction (Hart, 2005). As a consequence of its structural simplicity and multitude of behaviors, *C. elegans* has therefore become a model system for learning about more complex organisms.

Recently, the *Si elegans* platform was created to completely virtualize the nematode and its environment and thus reveal the links between the neural events that encode behavior (Blau et al., 2014; Petrushin et al., 2016). It is based on a high-performance FPGA-based computational *C. elegans* emulation architecture. The goal of the *Si elegans* platform is to serve as a tool for the understanding of the neural basis of behavior in *C. elegans*.

At its core, and as the main user interaction point, the platform provides an integrated web-based Graphical User Interface (GUI) framework for defining neural and network properties, configuring behavioral experiments and visualizing generated results. The GUI tool suite is available online to the scientific community (Si elegans Consortium, 2018c) and its source code has been made available at (Si elegans Web GUI tools Authors, 2018). This paper reports the implementation and functionality of the integrated GUI tools within a unified web platform, and describes the logical flow that guides the user through the experiment definition and results visualization process.

The following principal GUI components are presented in detail: (i) behavioral experiment definition GUI; (ii) neuron model design GUI; (iii) *C. elegans* neural network configuration GUI and (iv) results visualization GUI. In addition, the paper describes the implementation of exportable neural network NeuroML XML and behavioral experiment XML definitions that facilitate interoperability with other simulation environments. The paper reports the outcome of a user experience and tools acceptance study.

## 2. Materials and methods

This section specifies the graphical interaction principles and details the underlying technologies and methods employed in the implemented interfaces. Experiment definition includes the behavioral experiment description, the virtual neuron model capture and the neural network configuration. The results visualization displays the worm's locomotion and internal neuron variable traces to the user.

**Figure 1:**
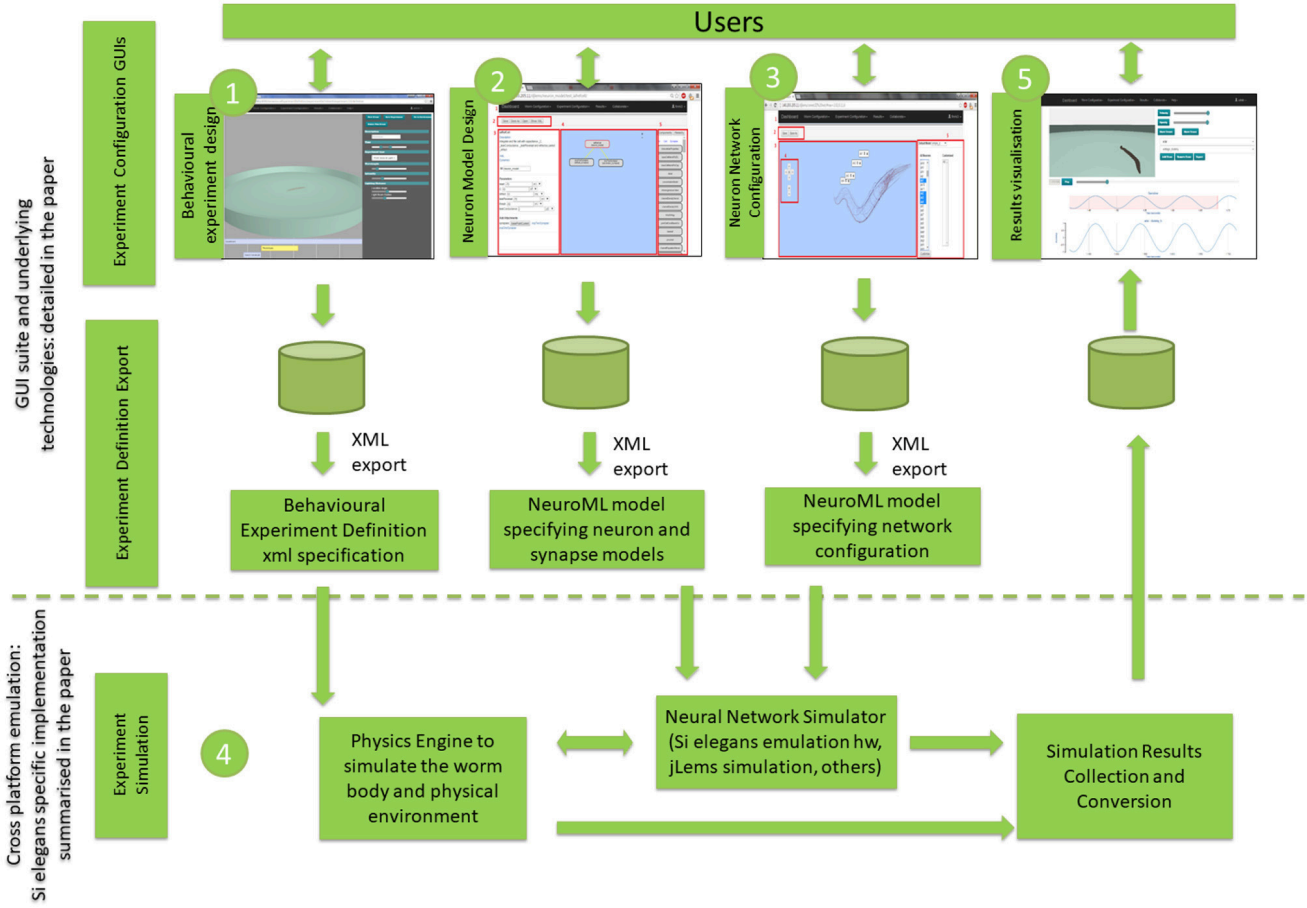
Overview of the proposed GUI toolsuite and underlying technologies within the global *C. elegans* simulation. The numbering reflects the typical workflow from graphical simulation definition to simulation execution and results visualization. GUI suite and underlying technologies block is detailed in the paper, while cross-platform emulation is summarized for the *Si elegans* specific implementation, providing references to papers with more detailed information.

Figure 1 illustrates the three experiment definition GUIs (Behavioral Experiment Design, Neuron Model Design and Neural Network Configuration) and the results visualization GUI. Figure 1 also depicts the GUI export functions and the experiment simulation process. Interaction with the experiment simulation is not possible during its execution. Results from the neural network and behavioral simulation are uploaded to the cloud for viewing and analysis.

In the Behavioral Experiment Design GUI, the user batch-defines the characteristics of the environment (e.g., shape of the experimentation plate, ambient temperature or obstacles placed in the plate), worm starting parameters (e.g., initial position and orientation) and the stimuli that the worm will receive during the experiment (e.g., touch or temperature changes). In the Neuron Model Design GUI, the user can create new neuron models using a building block editor, which contains the NeuroML2 CoreType Library defined in the Low Entropy Modeling Specification (LEMS) language (Cannon et al., 2014). In the Neural Network Configuration GUI, the user places and parameterizes neuron and synapse models in a graphically represented *C. elegans* connectome. The experimental design interfaces provide a machine-readable export format, which enables various simulation systems (such as the *Si elegans* platform Blau et al., 2014, jLems jLEMS, 2018, or pyLEMS Vella et al., 2014) to import this specification for simulating the defined *C. elegans* experiment. In the Results Visualization GUI, the user is can visualize experiment's resulting neuronal activity, worm locomotion and their combined mapping.

Elements depicted in Figure 1 within the experiment simulation block and their data exchange protocols are not the target of this paper, therefore they are not discussed in detail in the paper. However, to aid the reader's understanding, a summary of the specific implementation of the experiment simulation block within the *Si elegans* project is reported in the following.

In the *Si elegans* project, neuron and neural network simulation is performed on reconfigurable high-performance Field Programmable Gate Array (FPGA) hardware (Machado et al., 2015), while the worm's body, behavior and interaction with the environment is virtually emulated in an online physics engine (PE). The virtual worm emulates the shape, body-physics and muscular structure of the organism (Mujika et al., 2014).

Within the *Si elegans* specific implementation, transduction processes from behavioral experiment defined interactions or environmental stimuli (e.g., how are mechanical collisions detected in the PE fed back to sensorial neuron/muscle models) are described in Mujika et al. (2017). The neuron model and neuronal network configuration transformation from LEMS to FPGA-based hardware setup is described in Krewer (2016, Ch. 2).

Activity data for each neuron and traced variable is continuously transferred from the simulation framework to the database on the local server, while physical environment simulation locomotion data is transferred to the cloud for later reproduction on the web. More information on how the locomotion of the worm is processed in the Physics Engine (PE) server and then transferred for its visualization on the Results Visualization GUI is given in the Supplementary Data Sheet 1.

FPGAs communicate with the PE over a point-to-point 10 gigabit ethernet connection. Timestep length in the physics engine and FPGAs is configurable, as is the synchronization between both parts, in order to achieve real-time simulation. Communication protocol is detailed in Machado et al. (2015). The *Si elegans* platform grants single-user emulation due to its underlying computational hardware infrastructure (i.e., one FPGA per neuron approach Machado et al., 2015). Within the *Si elegans* project, the logic required for the blocks identified as “Experiment Configuration GUIs” and “Experiment Definition Export” are deployed as cloud services that are scalable with the number of users.

### 2.1. Technologies and tools

This section introduces the core technologies involved in the GUI tool suite. The GUI tool suite has been designed and developed with web technologies that rely on scalable cloud services (Epelde et al., 2015b). The use of web technologies ensures ubiquitous public access to services through standard web browsers.

The webserver employs the Django web-framework and Python programming languages. To avoid the need for installing client-side plugins, the developed GUIs use several available JavaScript libraries. HTML5, CSS, AJAX, and JavaScript are used for the user interface elements. The web page and all of its components (buttons, menus, etc.) are styled through the Bootstrap framework (Bootstrap, 2018). Web 3D applications were integrated into the behavioral experiment definition GUI, the network configuration GUI and the simulation results visualization GUI, making use of WebGL technology (WebGL, 2018). The specific JavaScript libraries for each GUI are described in the *Web-based GUI Toolsuite Implementation Result* section.

### 2.2. Behavioral experiment definition

This section analyses reference interoperability standards in biology and the strategy followed for encoding *C. elegans* behavioral experiments, together with graphical interaction principles followed for the GUI implementation.

#### 2.2.1. Input encoding strategy for *C. elegans* behavioral experiments

The Minimum Information About a Simulation Experiment (MIASE) guidelines ensure that experiments can be the unambiguously reproduced (Waltemath et al., 2011a). According to rule 2B of MIASE, all information needed for the correct implementation of the necessary simulation steps must be included through precise descriptions or references to unambiguous information sources. Behavioral input encoding is required for projects such as OpenWorm (Szigeti et al., 2014) or *Si elegans* (Blau et al., 2014).

With respect to simulation procedures, the Simulation Experiment Description Mark-up Language (SED-ML) provides a standardized, machine-readable format (Waltemath et al., 2011b) for the information required by MIASE guidelines to enable the reproduction of simulation experiments. Besides model identification, the MIASE guidelines provide for a precise description of simulation steps with all of the required information for obtaining numerical results to be reported in scientific publications. In SED-ML documents, the simulation experiment input is defined at a low parametric model level because the biological elements or subsystems in the used models (defined in e.g., SBML, CellML, or NeuroML) expect low-level signals (e.g., a voltage). In contrast, behavioral stimuli input for a living-organism (e.g., touch, chemical drop) operates at a higher abstraction level, which would need to be broken down and converted to computable variables before becoming suitable for an *in silico* simulation. It is therefore not well suited for defining the behavioral experiment input of a living organism in an *in silico* simulation. Other common tools to perform behavioral experiments, such as Precise (Neurobehavioral Systems, 2018), are usually used by psychologists for neurobehavioral research on humans (Mueller and Piper, 2014). However, there is no direct method to exploit these approaches in living organisms' *in silico* experiments.

Within the reported work on interoperability and standardization of computational neuroscience and systems biology research areas (Waltemath et al., 2011a,b; Mueller and Piper, 2014; Neurobehavioral Systems, 2018, see Epelde et al., 2015a for a more in depth state-of-the-art analysis), no specific solution exists that tackles the behavioral experiment input encoding in general or specific to the *C. elegans* nematode.

The definition of the *C. elegans* behavioral experiments input encoding has been inspired by Wormbook (Hart, 2005) behavioral experiments and by recent *C. elegans in vivo* research reports (Gabel et al., 2007; Ward et al., 2008). Gabel et al. (2007) and Ward et al. (2008) cover behavioral responses to behavioral input types that are not yet included in the WormBook. The developed encoding strategy comprises the *C. elegans* environment and experiment configurations. The behavioral experiment is structured by duration-based behavioral experiment types at the top level. The following three behavioral experiment types have been defined: (i) interaction at a specific time *t*; (ii) interaction from *t*0 to *t*1; (iii) experiment-wide configuration.

**Figure 2:**
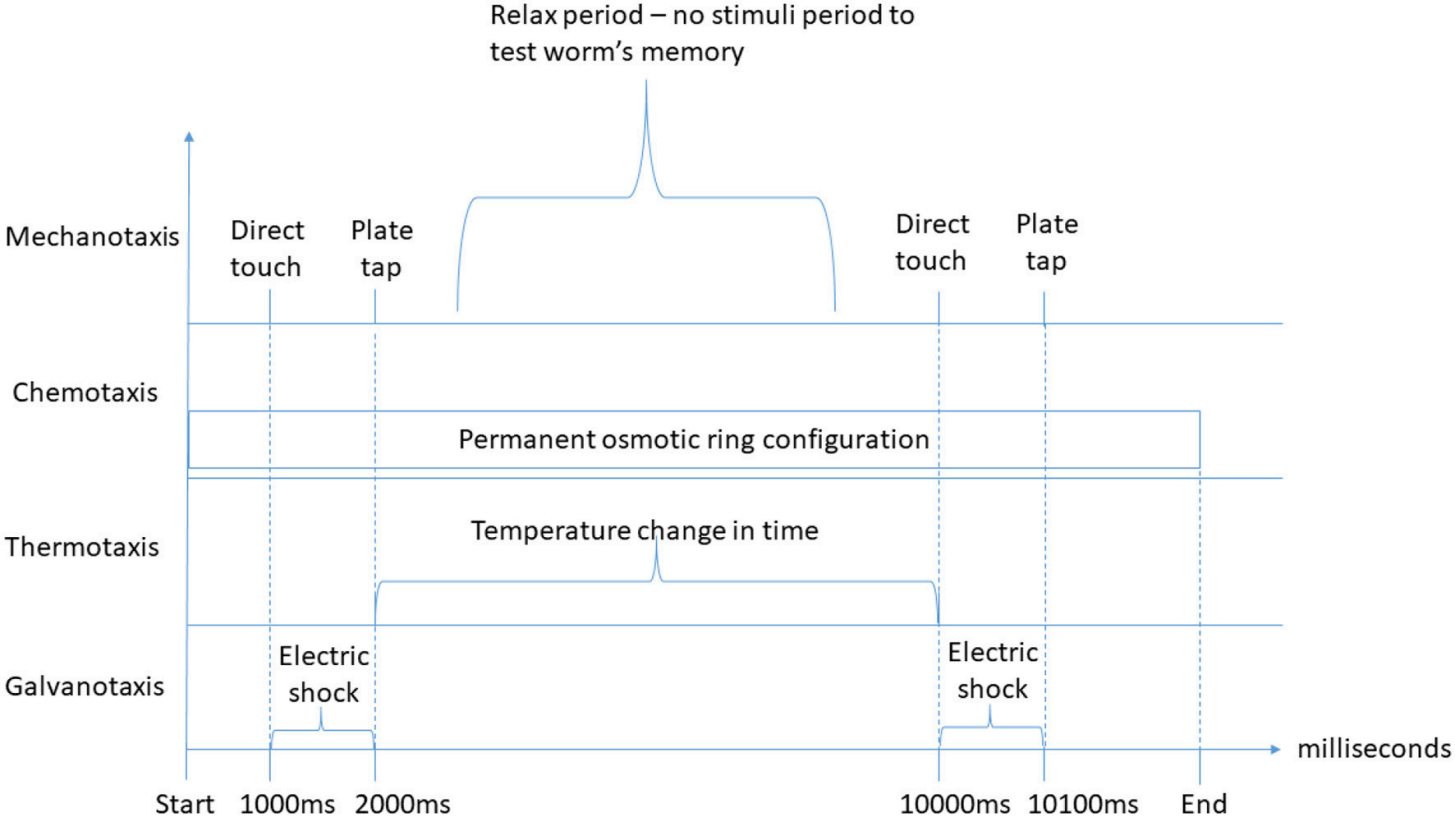
Duration-based behavioral experiment stimuli classification examples. On the top of the graph, direct touch and plate tap mechanotaxis stimuli of the “interaction at specific time” type are represented twice, separated by a period without stimuli, for instance to test the worm's memory. Below, a permanent osmotic ring configuration (chemotaxis) of the “experiment-wide configuration” type is represented, which is defined from the experiment start until its end. Further below, a temperature change over time of the “interaction from *t*0 to *t*1” type for thermotaxis experiments is defined. Finally, two electric shock galvanotaxis stimuli of the “interaction from *t*0 to *t*1” type are represented to illustrate the possibility of presenting different types of duration-based stimuli simultaneously. An earlier version of this figure was originally presented in Epelde et al. (2015a).

Each duration-based experiment allows for the definition of one or more interactions of each type. For each duration-based experiment type, each of the behavioral input types (i.e., mechanotaxis, chemotaxis, thermotaxis, galvanotaxis, and phototaxis) as identified by Hart (2005), Gabel et al. (2007), and Ward et al. (2008) is supported. Behaviors reported by Hart (2005), which are not driven by sensory behavioral inputs (i.e., feeding, egg-laying, mating, reproduction or defecation), have not been considered. Figure 2 depicts an example of the duration-based behavioral experiment classification defined at the top level.

**Figure 3:**
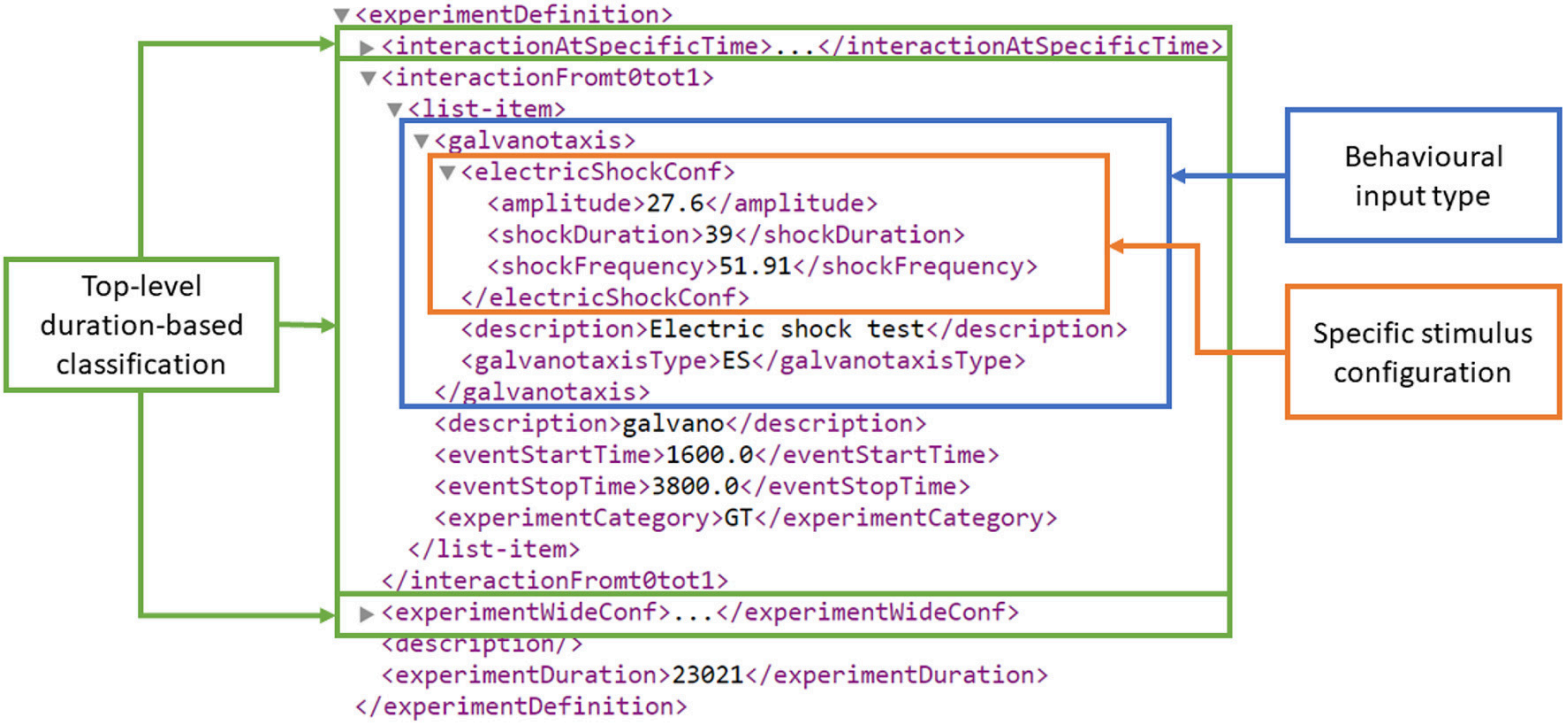
Experiment definition section of an example behavioral experiment input encoding XML. At the top of the XML hierarchy (figure bottom), *experimentDuration* defines the experiment's duration in milliseconds. The three top-level duration-based classification elements are highlighted in green, i.e., *interactionAtSpecificTime, interactionFromt0tot1* and *experimentWideConf*, corresponding to an interaction at a specific time *t*, an interaction from *t*0 to *t*1 and an experiment-wide stimulus configuration. The *interactionFromt0tot1* element contains a *galvanotaxis* behavioral experiment type and the duration-related stimuli start and stop time definitions (i.e., *eventStartTime* and *eventStopTime*). The *galvanotaxis* behavioral experiment type contains a specific electric shock stimulus definition for the amplitude (in nA), duration (in ms) and frequency (in Hz) under the *electricShockConf* tag.

At the third level, the concrete behavioral experiment inputs are defined as an XML schema developed in Si elegans Consortium (2018a). Figure 3 presents an example experiment definition section of a behavioral experiment input XML that contains multiple interaction instances.

**Figure 4:**
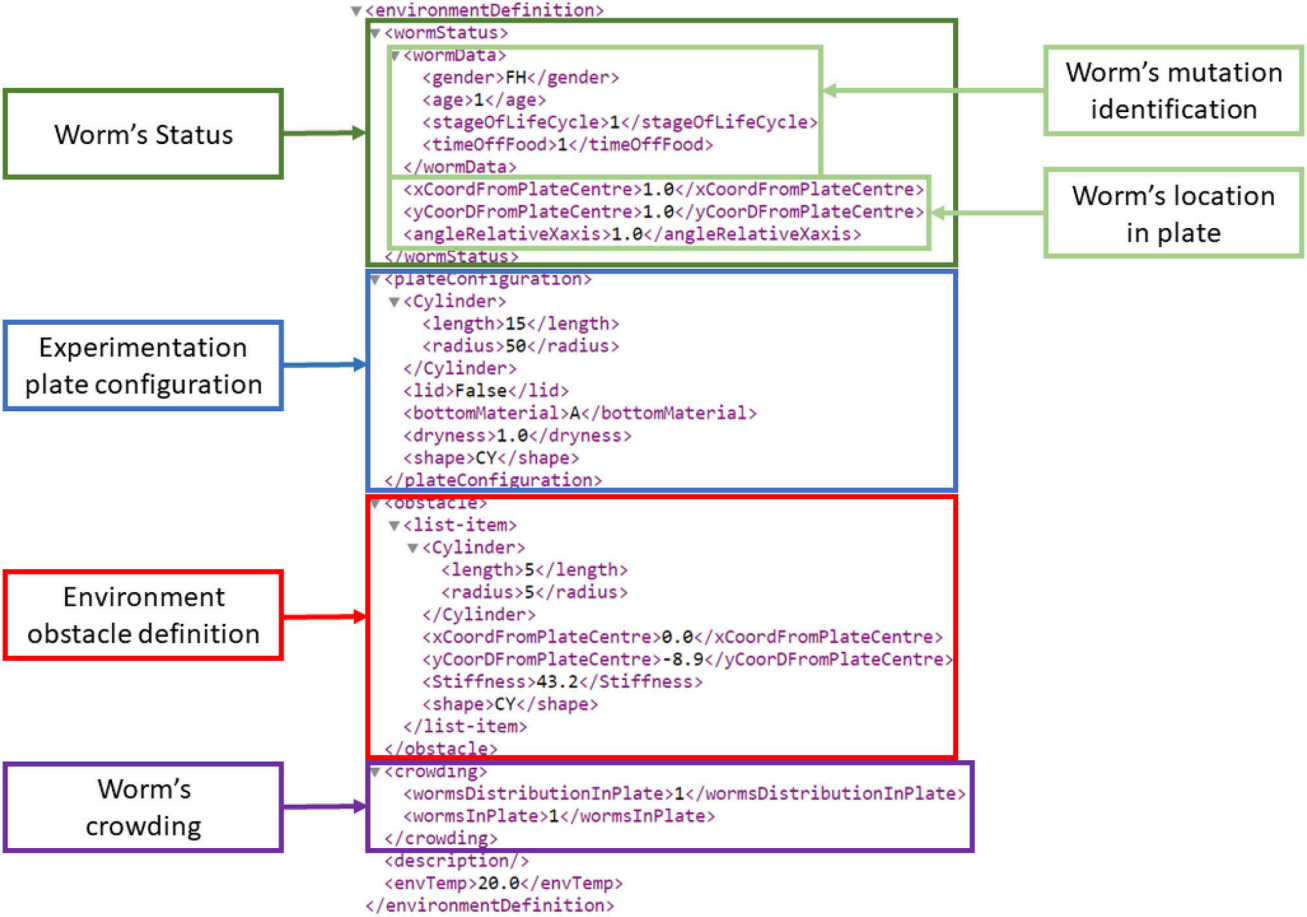
Environment definition section of an example behavioral experiment input encoding XML. On top, all worm-related information is defined (dark green frame). Within this worm status information, two light green boxes identify the worm mutation (*wormData*) and the worm's location on the experimentation plate. *wormData* identifies a one day old female hermaphrodite, at the *L*1 stage being 1 hour without food. Marked in blue, the experimentation plate configuration defines a cylindrical plate shape (border height: 15 mm; radius: 50 mm), an agar substrate (described as A), the plate dryness (1 mg over the plate's dry weight) and the lack of a lid. The red colored rectangle identifies a cylindrical obstacle (height: 5 mm; radius: 5 mm; stiffness: 43.2 N/m) located at defined distances and angle with respect to the central *X* and *Y* axes). The purple frame termed “worm's crowding” defines the number of worms on the plate (one in this case) and their distribution. The latter is represented by an index that points to predefined formulae.

The environment configuration structure and elements were extracted from the WormBook experiments review (Hart, 2005). The environment configuration is composed of the worm's mutation identification, crowding, location, plate and obstacles configuration, all of which were parameterized. Currently, the developed GUI tools and the *Si elegans* simulation framework make use of the worm's location and of the obstacle definition information only. The other elements were nevertheless included with more advanced simulator implementation in mind. Figure 4 presents an example environment definition section of a behavioral experiment input XML that describes the experiment environment together with the worm details. A complete behavioral experiment input encoding example is provided in Si elegans Consortium (2018b).

#### 2.2.2. Graphical interaction principles

Most traditional neural simulation software, such as NEURON (Carnevale and Hines, 2006), BRIAN (Goodman and Brette, 2008), or GENESIS (Bower et al., 2003), allows the definition of stimuli at the neuronal level, either through script files, graphical user interfaces (GUIs) or via a command shell. For instance, the web-based Geppetto simulation platform (Geppetto, 2018) makes use of a script-file-based stimuli definition. While most GUI-based stimulus definition environments are limited to parameterizing the to-be-injected current only, the GUI of the AnimatLab framework (Cofer et al., 2010) includes a 3D visualization of the animal's inner and outer body elements, which can be selected to enter stimuli (mechanical and neural) through an element-specific dialog. Following this example and the recent trend of augmenting realism in e.g., medical simulation frameworks (Allard et al., 2007), we combined different graphical interaction principles for the behavioral experiment definition. These include a 3D visualization of the animal and its experimentation environment, a timeline for visualizing and selecting the behavioral inputs, and a sidebar for their tuning, using edit or add boxes, buttons or sliders.

### 2.3. Neuron model design and *C. elegans*-specific network configuration

This section analyses existing neuron computational model approaches and describes the chosen encoding strategies for both the *C. elegans* neuron model description and the neural network configuration.

The creation of computational biological neuron models and neural networks is an ever-growing field of research. Various model specifications have been developed (Abbott and Kepler, 1990; Izhikevich, 2003; Burkitt, 2006; Brette et al., 2007). A number of *C. elegans*-specific connectomes have been reported (Durbin, 1987; Varshney et al., 2011; Jarrell et al., 2012). Computational models are essential tools for verifying and testing our understanding of biological processes, including biological neural networks. Models in computational neuroscience are becoming increasingly available due to efforts by model repositories such as ModelDB (Hines et al., 2004) and the Open Source Brain repository (Open Source Brain, 2018). The majority of neuron and neural network models are implemented in simulator-specific formats (such as NEURON Carnevale and Hines, 2006 and BRIAN Goodman and Brette, 2008), or as part of custom purpose-built simulators in programming languages such as C or Java. This can make it difficult to reproduce computational simulations without the specific simulator setup or the correct version of each software package (Gleeson et al., 2010). This difficulty highlights the need for reproducible simulator-agnostic and easily understood modeling specifications and has led to the development of the NeuroML (Gleeson et al., 2010) modeling language. The NeuroML language contains specific building blocks for the construction of neuron models and networks. This makes the language easy to understand and use for domain experts. NeuroML relies on each compatible neural simulator implementing a small set of standard components of standard models. However, some simulators implement basic neural building blocks such as ion channels differently. As such, NeuroML-defined models are still not completely reproducible in any simulator.

#### 2.3.1. LEMS and NeuroML as an export format

The Low Entropy Model Specification (LEMS) (Cannon et al., 2014) was developed to codify how neuron models and basic neural components should be implemented in a simulator. LEMS is a declarative language and functionally describes neural components, neuron models and entire neural networks. A library of LEMS neuron model descriptions exists for all components used by the NeuroML2 neural network description language. Prior to simulation, LEMS descriptions are often exported to various high-performance software simulators such as NEURON and BRIAN for speed-optimized execution.

The portability of LEMS, along with its machine readability, makes it an ideal interchange format, not only between simulators, but also from model design environments. LEMS separates the descriptions of model dynamics from parameter values, with model dynamics described in ComponentType elements. A ComponentType contains model dynamics in the form of event-driven and time-driven variable changes. All ComponentTypes must be parameterized once instantiated as Components. Consequently, a single ComponentType may describe multiple different behaviors depending on how it is parameterized. The Izhekevich neuron model (Izhikevich, 2003) is an example of a model for which one set of model dynamics translates to radically different behaviors depending on its parameterisation. This separation of dynamics from parameters results in a high degree of reusability of model designs.

The Neuron Model Design GUI uses the NeuroML2 CoreType Library (Cannon et al., 2014) components as building blocks for assembly into neuron and synapse models and consequent parameterisation. Once a model is completed, the resulting NeuroML Component instances are combined with LEMS ComponentTypes and exported as a LEMS XML file.

#### 2.3.2. Graphical interaction principles

The development of computational biological neuron models can be performed using simulator-specific formats, more-general programming languages, or more standard targeted markup-based modeling languages. The development of the models is usually supported by text editors, integrated development environments or markup language editors. Neural model editors (or generic editors used in such tasks) with a GUI do not offer much visual aid beside schema and syntax validation support. From a visual interaction perspective, most advanced approaches include the use of standalone graphical design-based modeling tools (MATLAB Simulink and NI LabVIEW), which exploit existing function blocks to construct mathematical and logical models and process flow for neuronal model definition (Kullmann et al., 2004; Zhang et al., 2009).

In our neuron model design GUI approach, we have implemented a building blocks-based drag and drop, connection and parameterisation concept (similar to graphical-design-based modeling tools) as a web tool, implementing NeuroML2 Coretype Library components as building blocks. In this way, a multiuser graphical design-based neuron modeling tool is provided, which through NeuroML2 enables exporting of LEMS descriptions, supported in many neuron simulation software. The model parameters and neuron equations are validated by the LEMS parser.

Many existing neural network configuration tools allow configuration of the number of neurons and their connections using GUI tools (Yoshimi, 2008; Cofer et al., 2010; Neuroph Project, 2018). For instance, AnimatLab (Cofer et al., 2010) enables graphical drawing of connections between neurons and the addition of synapses.

The focus of our development was to cover the known *C. elegans* connectome (Varshney et al., 2011) with a fixed network topology. *C. elegans*-specific neurons or synapse can be selected and models assigned. To ease the identification of such neurons, a simplified version of the OpenWorm 3D worm browser (OpenWorm, 2018) has been developed. GUI interaction allows definition of a generic neuron and synapse model for the whole worm, followed by selection of neurons from a list, or from the 3D worm diagram, and customization of neurons with specific models.

### 2.4. Results visualization

This section analyses previous work on *C. elegans* simulation-related result visualization of individual neuronal activity, worm locomotion and their combined mapping. Based on the state of the art analysis, the joint results visualization strategy is described.

#### 2.4.1. Neuronal activity visualization

The development of advanced visualization tools for exploring brain activity is an active research field. Previous work has been identified and analyzed to inform the definition of a *C. elegans*-optimized visualization tool.

For instance, in Mulas and Massobrio (2013) 2D tools provide different information about neurons such as type (inhibitory or excitatory), potentials, connections, spiking rate, etc. The neural network interconnectivity can also be depicted, yet lacks information on neural morphology. Sousa and Aguiar (2014) rendered thousands of neurons by placing colored spheres in a 3D environment, each one representing a neuron at a certain potential.Although the use of colors has limitations as the number of neurons rises, this concept has been used in as part of the *C. elegans* activity viewer.

The concept of the 2D connectome exploration graph tool presented by Bhatla (2018), has been used to visualize pre-and postsynaptic neurons, where a selected neuron is surrounded by the neurons that are related or connected to it.

The OpenWorm initiative uses different, somewhat complementary approaches to visualize neuronal activity. The first approach renders the complete anatomy of the worm in a WebGL based web page (OpenWorm, 2018). The user highlights different parts of the worm (including neurons) in order to investigate anatomy and connectivity. However, neuronal activity cannot yet be visualized. The second approach uses hive plots for the connectome visualization (Tabacof et al., 2013), i.e., plots where neurons are grouped on radially distributed linear axes, and relations are drawn as curved links. A third approach is embedded in the main platform of the OpenWorm project (Geppetto, 2018) and displays neurons as spheres together with additional information about their voltages in a line chart in an adjacent menu. Our work combines the morphology visualization of the first approach of the OpenWorm project and the activity visualization in a line chart of their third approach.

#### 2.4.2. Worm locomotion visualization

All of the reported models of the locomotion of *C. elegans* include a visualization engine. The pioneering methods operate in 2 dimensions (Niebur and Erdos, 1991; Suzuki et al., 2005). One of the most complete 2D systems (Boyle et al., 2012) includes complex neuronal simulation and muscular simulation. A zenithal view is set to follow the contour of the animal. However, the springs that represent the muscles are not shown.

In recent years, there have been several efforts to model the locomotion of the worm in three dimensions. One of the simplest is reported in Mailler et al. (2010), where the worm is represented with a row of cylinders that are interconnected using joints. It is not very different from reported 2D approaches since the cylinders are completely rigid. Another 3D system is presented in Palyanov et al. (2011) and has been used for chemotaxis experiment visualizations (Demin and Vityaev, 2014). An OpenGL application shows a plate with a worm moving on it. The cuticle is not shown, and only the muscles are displayed; specifically, a pair of cones represents each muscle of the nematode.

The 3D system presented in Palyanov et al. (2011) is the starting point of the OpenWorm project regarding the modeling and visualization of the locomotion of *C. elegans*. (Vella et al., 2013) uses a physics engine that works with Smoothed Particle Hydrodynamics. The resulting positions of all of the particles that comprise the worm and its environment are shown in an OpenGL graphical application. In order to take this simulation to the web, Geppetto, the web platform developed in the OpenWorm project, renders simple 3D deformable objects that are modeled by particle systems. Nevertheless, a 3D reproduction of the locomotion as the result of the worm simulation has not yet been achieved.

#### 2.4.3. Graphical interaction principles

The small number of neurons that are studied in assays with *C. elegans* permits the implementation of different approaches to those presented earlier, in particular the provision of a representation of neurons in the 3D environment with their authentic shape, placement and connectivity. Furthermore, those neurons should follow the physical movements of the whole worm. Thus, the effect of the interaction with the environment (collisions, sensing new chemical substances, etc.) could be observed *in situ*. The core of the results visualization GUI tool presented in this paper is the simultaneous web visualization of a 3D reproduction of the locomotion results and of the driving neuronal activity using biologically accurately placed neurons.

Along with the combined 3D worm locomotion and neural activity visualization, a timeline indicates which part of the simulation is being shown, in order to help in the in-depth exploration of the neural processes and their consequent physical behavior. The user can switch from neuron activity graphs to behavioral experiment input definition, and vice versa, allowing the exploration of causality between received stimuli, neural function and resulting locomotion. Play/pause functionality and navigation to a specific time by dragging the slider control support interact with the simulation results, and allow the user to explore the simulation at any pace.

## 3. Web-based GUI tool suite implementation result

This section presents the integrated web-based GUI tool suite implementation. Further detail and example use cases for each interface are provided in Supplementary Data Sheet 2. The results of a usability study are presented and discussed.

### 3.1. Behavioral experiment definition GUI

**Figure 5:**
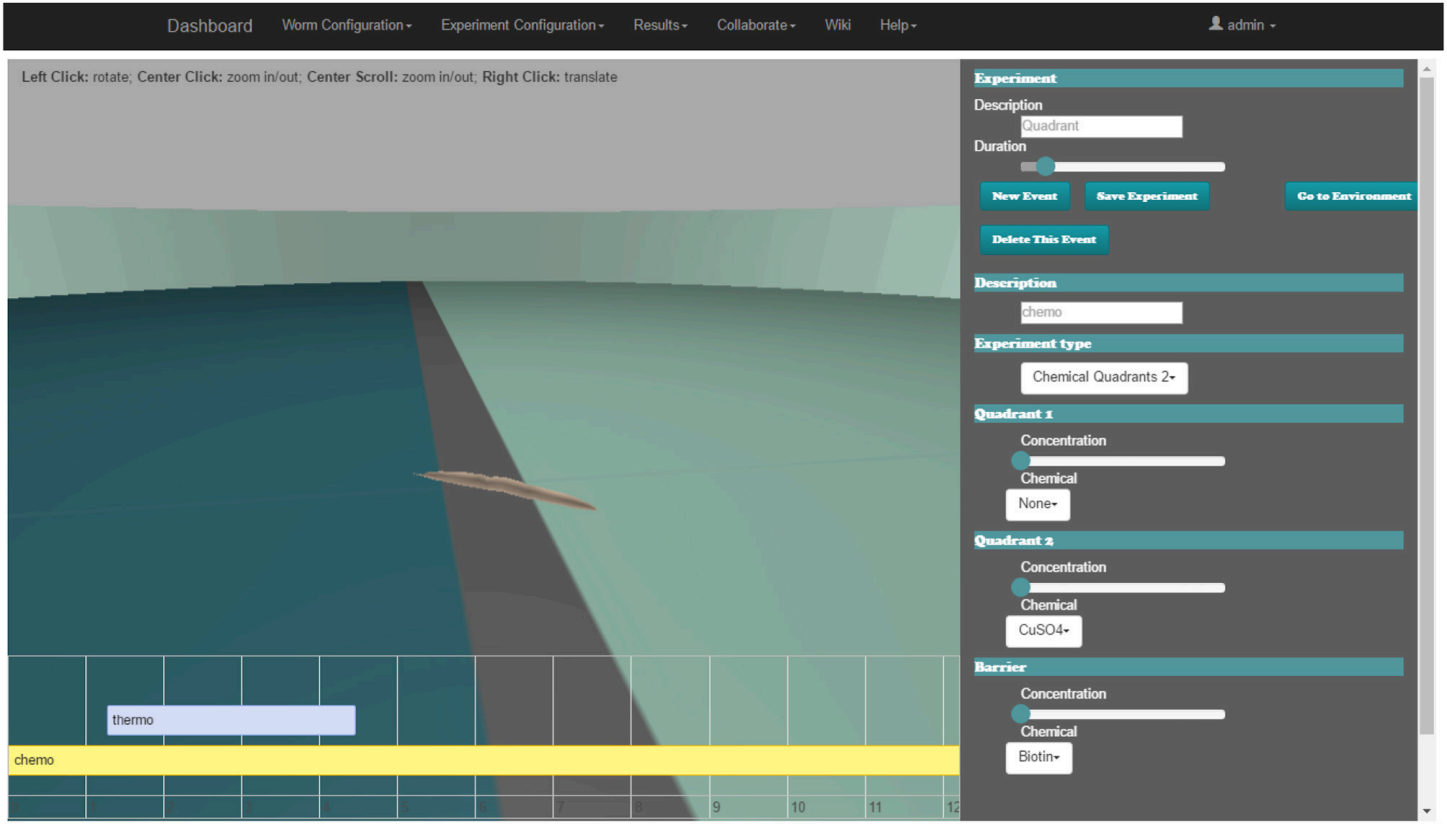
Web-based *C. elegans* behavioral experiment definition GUI for configuring behavioral input parameters. 3D window (middle), experiment definition window (bottom) and the properties window (right).

The web-based behavioral experiment definition GUI is used to define all aspects related to the *C. elegans* simulation experiment. The GUI (Figure 5) includes three main windows; the experiment definition window, the properties window and the 3D window.

The experiment definition window includes a timeline editor (lower area of the web-page). All of the events which happen during the assay (events occurring at a precise time, in intervals or permanently) are placed on the experiment configuration timeline. Events follow the input encoding policy for behavioral experiments introduced in the *Input Encoding Strategy for C. elegans Behavioral Experiments* section. The user can easily add a new event, move its position on the timeline or change its duration (if it is an interval).

When a new event is added or an existing one is selected, all of its properties are displayed in the properties window (on the right side of the browser). For example, a mechanosensation event can be defined, where the worm is touched over a certain period with a force of defined strength using a small eyelash widget at a specified body location. Another event configuration is the application of a drop of a selected chemical onto the plate at a specified location.

The user can activate the environment properties definition window (replacing event properties in the properties window) to specify all of the parameters of the worm habitat. Some parameters are discrete; they are defined with a drop-down menu, e.g., the shape of an obstacle to be inserted in the environment or the substance that surrounds the worm. Other parameters are continuous and are defined with a slider, e.g., the initial position and orientation of the worm.

In the 3D window (center of the browser), the starting point of the simulation (obstacles, plate shape, position of the worm, etc.) is rendered using the Three.js library (three.js, 2018) that makes use of WebGL (WebGL, 2018). The user can explore the experiment arena with the mouse to define the properties of the environment or the experiment.

A client-side framework based on the model-view-controller software architectural pattern has been used (ember.js, 2018). Transitions and changes that happen in the browser are easily controlled without changing the url, taking advantage of AJAX technology to avoid reloading complete pages, thus reducing bandwidth requirements.

The Supplementary Video 1 demonstrates a web-based behavioral experiment definition GUI.

### 3.2. Neuron model design GUI

This section describes the Neuron Model Design web GUI, which defines and links a group of neuron model component building blocks. Each component, described by the ComponentType definition, represents an element that contributes to the neuron model response. A component may export variables to other elements of the neuron model, execute its own model response, generate events, or act upon events generated by other components within the neuron model. The Neuron Model Design web GUI contains all of the components from the NeuroML2 CoreType Library. This enables the definition of neuron models using (Hodgkin and Huxley, 1952) format gating variables and ion channels, as well as numerous common point neuron models such as Integrate and Fire and Izhikevich neuron models (Izhikevich, 2003).

**Figure 6:**
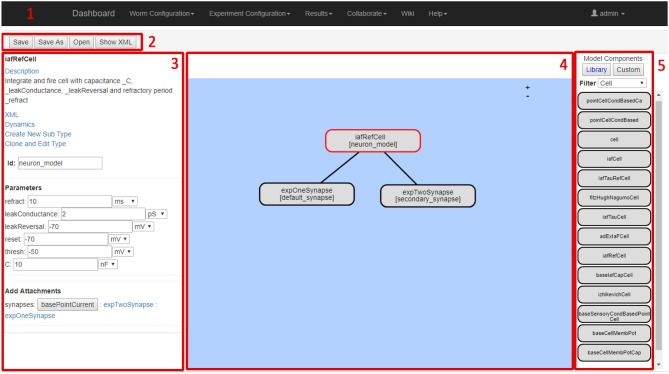
Neuron Model Definition Screen.

The screenshot in Figure 6 illustrates the Neural Network Configuration GUI. The GUI includes five main elements; the main navigation bar (1), the file functionalities buttons (2), the model details panel (3), the model definition workspace (4) and the component selection panel (5).

The model details panel presents the details of the currently selected component. The parameters of each component can be entered by the user. The model definition workspace panel enables drag and drop placement of model components to the current model. Clicking on a component causes its details to be shown and edited in the model details panel. The component selection panel allows users to browse and select from currently available model components. The selected model component details (name, description, dynamic behavior etc.) are shown in the left-hand-side model details panel.

Supplementary Video 2 demonstrates the Neuron Model Definition GUI usage.

### 3.3. Neural network configuration GUI

The Network Definition GUI is used to specify which model to apply in which neuron in the *C. elegans* network, and which synapse to use for each synaptic connection in the *C. elegans* network. This GUI is also used to customize the parameters in each neuron in the *C. elegans* network. Neuron variables for recording and analysis as part of the results visualization are automatically selected after the network configuration is complete.

**Figure 7:**
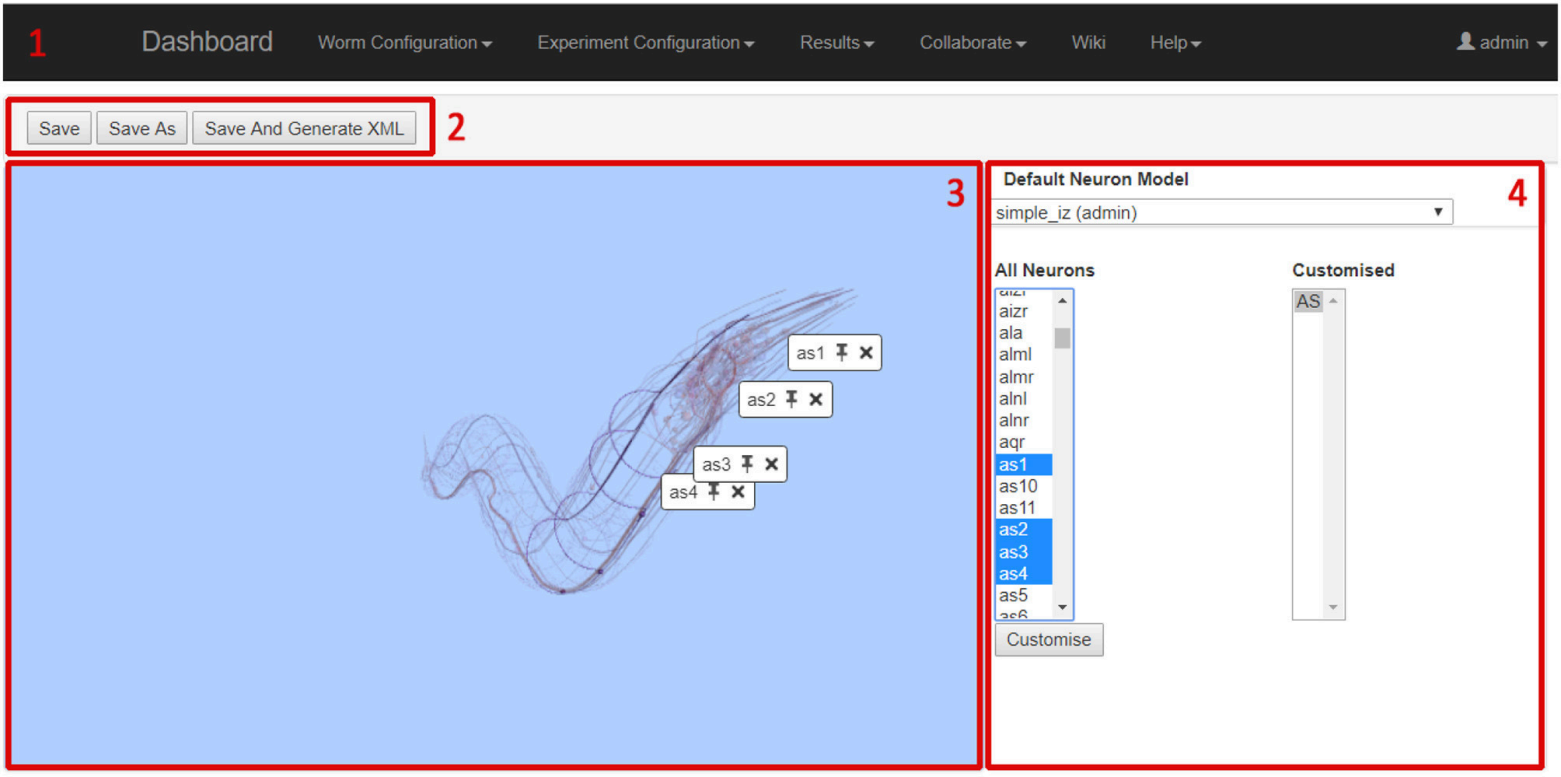
Neural Network Configuration GUI.

The screenshot in Figure 7 shows the Neural Network Configuration GUI. The GUI includes four main elements; the main navigation bar (1), the file functionalities buttons (2), the 3D network view (3) and the network details panel (4).

The 3D network view shows a 3D representation of all neurons in *C. elegans*. As neurons are selected by the user, they are highlighted and all other neurons are faded. This allows the user to browse for specific neurons and to see how neurons relate to each other in physical space. Note that the neural network being configured is composed of point neurons, and the morphology is only displayed to illustrate neuron position and connectivity. The 3D viewer is based on the Worm Browser which is part of the OpenWorm project (OpenWorm, 2018). The network details panel is the main working area for specifying the details of the network; the displayed data changes depending on the current activity of the user. The three main display modes (i.e., neuron selection mode, neuron parameter mode, and synapse selection mode) allow to select and parametrise neuron and synapse models to be used in the neural network configuration.

Supplementary Video 3 demonstrates the Neuron Network Definition GUI usage.

### 3.4. Results visualization GUI

This section describes the joint results visualization web GUI. After an experiment is defined and executed in a suitable simulator (for example the *Si elegans* hardware simulator or jLEMS simulator), the results are presented to the user in the results visualization web GUI. Simulation results can be separated into the physical motion results and the neuron variable traces.

In the implemented platform, the user can either use the joint visualization web GUI tool or an individual web GUI for visualizing each result type.

The web page developed for the behavioral experiment results visualization has three distinct parts:

- the worm visualization 3D window, where the recorded behavior of *C. elegans* is shown as a virtual representation of the worm.
- the controls window, where the 3D controls and the timeline visualization modes are selected (behavioral stimuli vs. neural traces). If neural traces visualization is selected, the user can also select specific neurons for result viewing.
- the timeline window, where (in neural traces visualization mode) variable traces of the selected neurons can be observed and (in behavioral stimuli visualization mode) the behavioral stimuli defined for the experiment can be observed. Both modes are synchronized with the worm locomotion in the 3D window.

**Figure 8:**
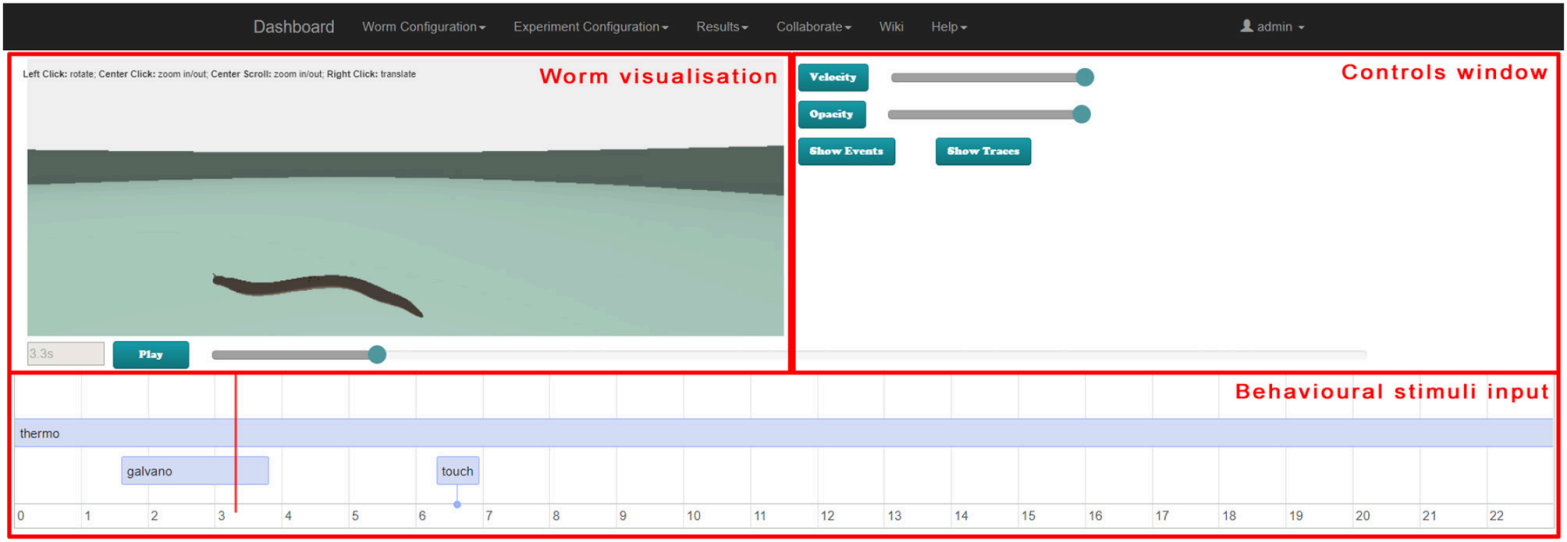
Web-based virtual worm simulation results visualization with behavioral stimuli input. The virtual worm visualization is synchronized with behavioral stimuli at the bottom and can be controlled by sliders on the right-hand side and Play / Pause button.

Figure 8, 9 illustrate the worm simulation results visualization in behavioral stimuli mode and in neural traces visualization mode consecutively. Supplementary Video 4 demonstrates Results Visualization GUI usage.

**Figure 9:**
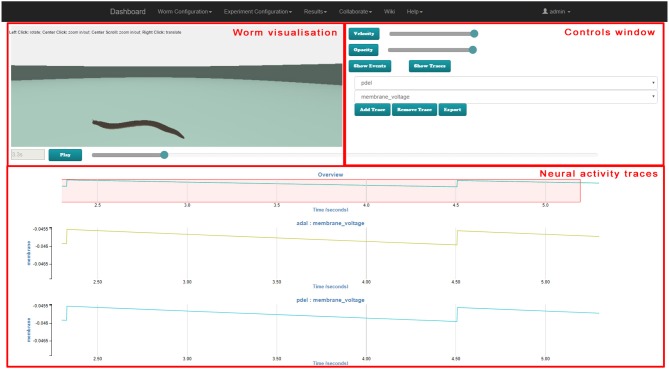
Web-based virtual worm simulation results visualization with neural traces visualization. The virtual worm visualization is synchronized with the neural activity at the bottom. Parameters can be selected in controls window on the right-hand side, and can be controlled by using the slider on the right-hand side and Play / Pause button.

The 3D window shows a 3D reproduction of the worm including its cuticle (skin). The opacity slider in the controls window enables changing of the opaqueness of the cuticle to render the simulation results analysis easier in those cases where the visualization of neurons is not desired.

Standard play and pause buttons in the timeline window control the visualization of the simulation. When the spiking of the neurons and its behavioral consequences occurs quickly, the visualization of the simulation results can be slowed down by a velocity slider in the controls window. Moreover, a specific simulation moment can be selected in the timeline window.

**Figure 10:**
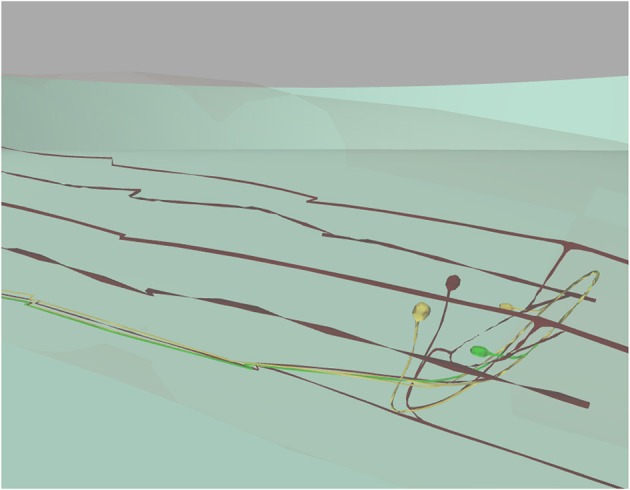
3D window focused on specific neurons visualization. 3D window with camera set to show a selected neuron (in green), two postsynaptic neurons (in yellow) and all remaining neurons in dark red below the worm's cuticle (almost transparent). This figure was originally presented in Mujika et al. (2015).

*C. elegans* neurons have different shapes, including very elongated ones. Neurons are not simple spheres, as presented in previous work (Sousa and Aguiar, 2014; Geppetto, 2018). Most of the *C. elegans* neurons are crowded in its head which makes the exploration of neurons in a 3D environment difficult. However, the developed results visualization enables selection of a specific neuron. This is highlighted in green and its postsynaptic neurons are highlighted in yellow (Figure 10).

Reproducing place and trace neurons realistically in a 3D environment is very important when exploring the neuronal response of the worm. For instance, in mechanosensation experiments, the user is able to focus on the zone where the worm is being touched, in order to observe which neurons are located in that zone and to track their activity.

In the 3D window, the three.js library (three.js, 2018) renders the virtual worm in WebGL. It loads and animates the 3D models, offers a mouse-guided control of the 3D scene camera position and direction, highlights different parts of the worm, handles transparencies, etc.

The vis.js library (vis.js, 2018) has been chosen for the event visualization in the timeline window. The dc.js library (dc.js, 2018) has been selected for neuron potential and neuron model variables plotting.

The ember.js library (ember.js, 2018), a client-side framework based on the model-view-controller software architectural pattern (with AJAX technology), controls the changes of the web page without reloading the entire page.

### 3.5. Acceptance evaluation

The web GUI tools for the virtual *C. elegans* neuron model and behavioral experiment setup and for the results visualization have been provided to the research community. This section presents the results of an acceptance study and an evaluation of the user experience of the tools.

The following functionality has been included in the web-based GUI platform after an initial user review:

- WormBase (Virtual Worm Project, 2018) neuron specific pages are referenced from the Neural Network Configuration UI. During neural network construction, the details for any neuron from WormBase may be directly accessed, helping the user to make decisions about their network configuration during network design and capture.
- Export has been enabled for behavioral experiment definition (following the *Si elegans* XML schema) and for neuron models (following the LEMS standard). A full LEMS export for neural networks is available so that a complete network simulation (without muscles) may be performed offline using tools such as jNeuronML and the NEURON simulator. A spreadsheet export of the simulation readback results has also been provided.
- A range of example models have been modeled, including their X, Y, Z positions. Usability features include (a) applying models to groups/subgroups of neurons/synapses, (b) exporting defined models and links to related *C. elegans* databases, (c) the use of putative neurotransmitters for synapse placement using the Virtual Worm connectome data.
- Separate locomotion results visualization and neuron results visualization GUI tools are provided.

#### 3.5.1. Community outreach and survey feedback

The tools and concept described in this paper have been demonstrated to users at the European Worm Meeting (Mujika et al., 2016). User opinions were captured with a focus on the usefulness of the implemented functions (Google form-based questionnaire) and included a user experience assessment survey (Attrakdiff 2 questionnaire Hassenzahl, 2004, 2018).

The Google form-based questionnaire included a free text entry to capture detailed feedback on specific GUI components. An opinion on proposed future functionality was obtained with a 5 level Likert scale (not important to very important). An additional free text entry was provided for suggesting other functions. A general usability section was included.

AttrakDiff has four dimensions for evaluating the system. The dimensions are pragmatic quality (PQ), the hedonic quality - identity (HQ-I) and stimulation (HQ-S) as well as the attractiveness (ATT). PQ captures whether the implemented functions are appropriate to achieve proposed goals. HQ-I measures the capability of a system to satisfy the human need to be perceived by others in a certain way. HQ-S captures the level of a product to fulfill the human need to improve personal skills and knowledge. ATT captures the general positive or negative assessment of the appeal of the system.

Each of the four dimensions has seven items representing attributes of the system. Within each item, a word pair spans a scale between two extremes. The scales consist of seven stages between the word-pairs. The oppositional word pairs consist of two conflictive adjectives like “conservative”–“innovative,” or “discouraging”–“motivating.”

35 users have registered on the *Si elegans* platform (Si elegans Consortium, 2018c) where the presented GUI tools are deployed). Twelve users responded to the acceptance evaluation questionnaires.

Forms and results of the Google form-based questionnaires are included as Supplementary Data Sheet 3 and Supplementary Table 1. Results of the user experience AttrakDiff measurement tool are provided as Supplementary Table 2.

#### 3.5.2. Acceptance evaluation discussion

The global GUI concept and developed tools were perceived positively, as intuitive and easy-to-use tools for experiment definition and visualization of result. Users pointed out the need for in-screen help for a smoother user experience. Most users supported the use of a web GUI tools *C. elegans* simulation environment, especially for testing different hypotheses before validating them through *in vivo* experimentation. A validation of the GUI tools together with the emulation platform was considered important to attract users. While users report that the developed GUI concepts could help in testing different hypotheses before validating them through *in vivo* experimentation, the delivery of a proxy virtual worm is still a matter of research. The GUI concepts presented in this paper, represent a specific subset of the required building blocks and functionality to reach that goal.

Regarding the usefulness of the developed concepts, the following results were obtained. Behavioral experiment definition GUI tool was valued as an appropriate visual tool to achieve the experiment configuration task. Most suggestions referred to speeding up some tasks and displaying more assistive information on utility usage. As for the neuron model design GUI concept, users valued it as organized and intuitive and judged positively on the drag-and-drop functionality and the ease of parameterising models. Regarding neural network configuration forms, users valued this GUI tool as easy to use, despite some users commenting on improving the 3D worm secondary neuron selection option for model assignment. With respect to results visualization, users valued the side-by-side viewing of locomotion and neural results and its intuitiveness. The time required to load the GUI (the implemented 3D worm model with the large detail of neurons required a quite large file) was considered long.

**Figure 11:**
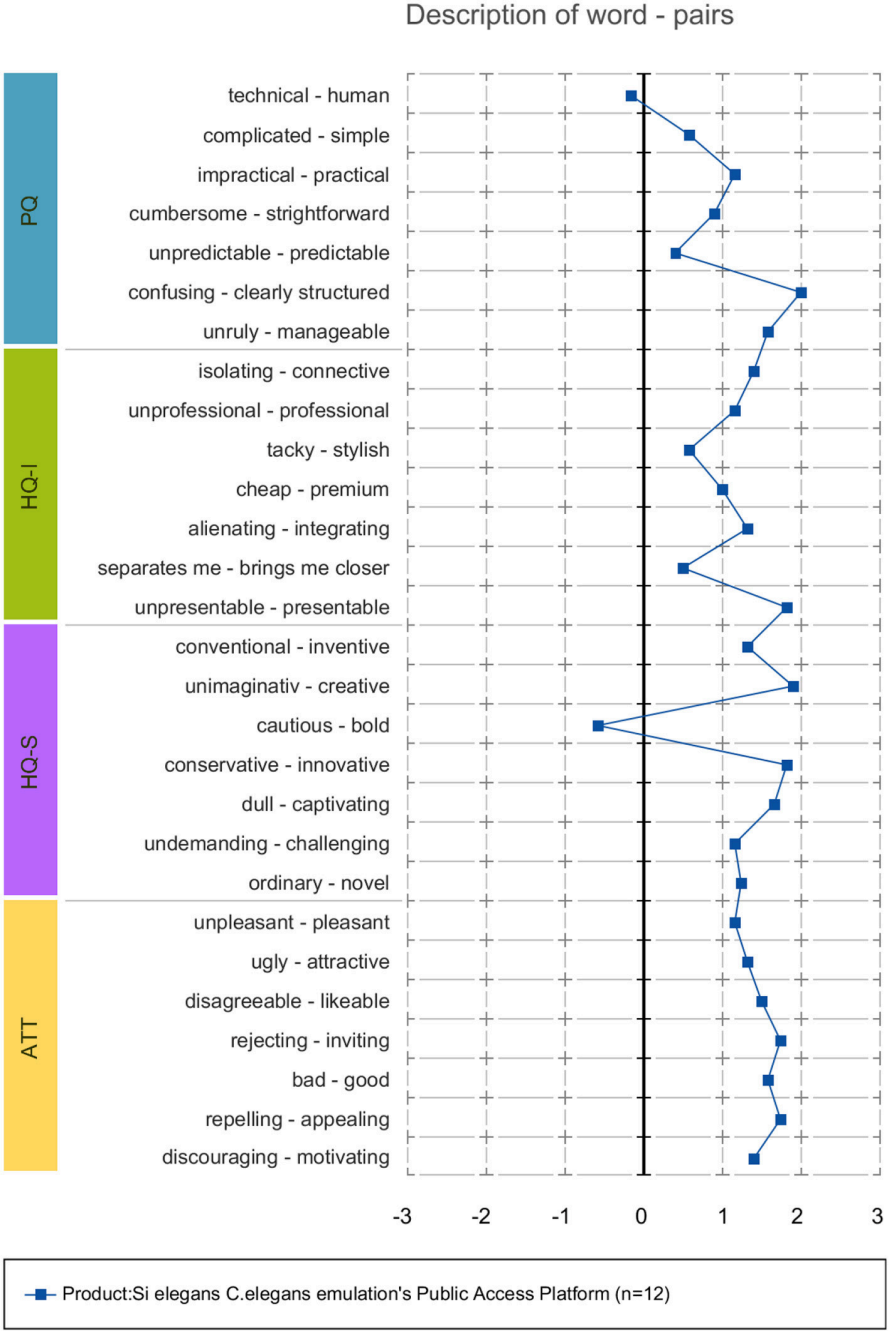
Mean ratings of word pairs.

**Figure 12:**
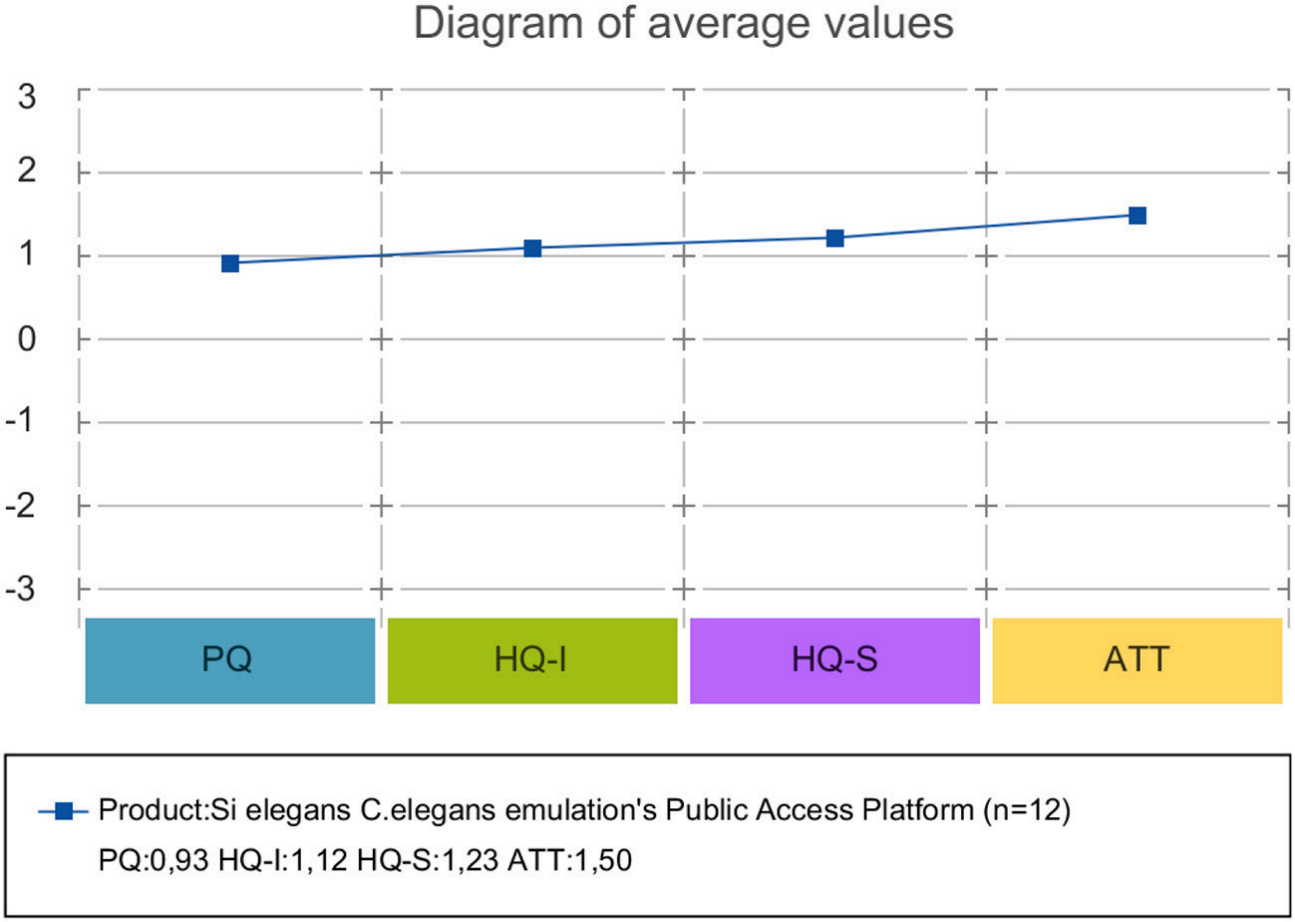
Mean value of all four AttrakDiff dimensions.

**Figure 13:**
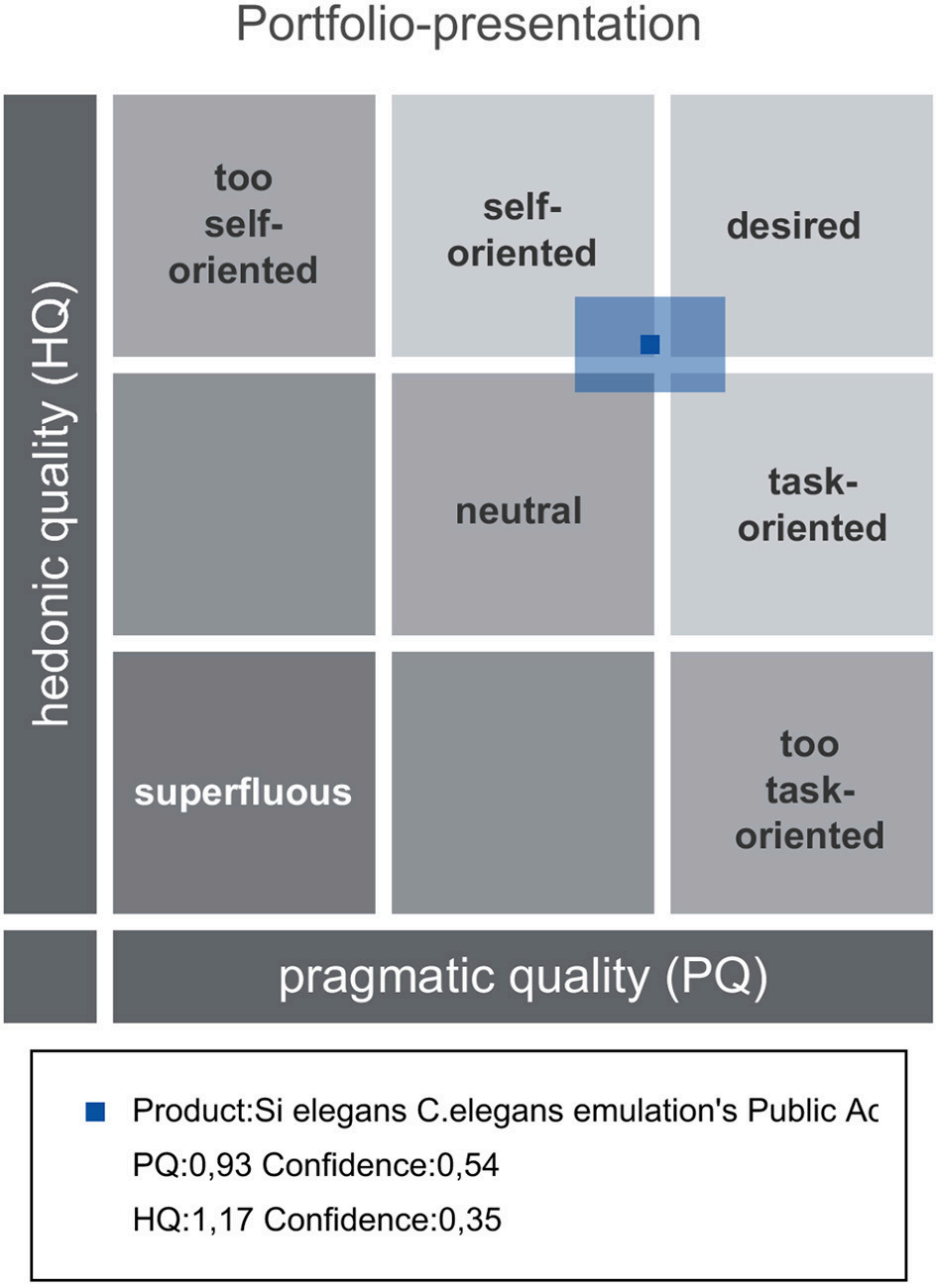
Results overview presenting PQ and HQ dimensions and the confidence rectangle.

Attrakdiff2 tool results for user experience evaluation pointed out the GUI tools as good, innovative and practical. Figure 11 depicts mean rating of word pairs and Figure 12 depicts mean values for the different Attrackdiff dimensions. This is in line with comments captured in Google forms where users defined the integrated web GUIs as clearly structured within the same web style-sheets and well-ordered according to their place in the logical execution of the emulation. Despite having good attractiveness, motivation and stimulation results, usability and usefulness dimensions of Attrakdiff2 show an improvement margin (Figure 13), which we interpret as the need to advance in the validation of the GUI tools within a complete *in silico* simulation framework, to achieve the proposal's adoption as part of a complementary tool to *in vivo* experiments.

A more detailed descriptive analysis of the acceptance evaluation survey results is provided in Supplementary Data Sheet 4.

## 4. Conclusions and future work

This paper presents a complete web-based graphical interaction tool suite for the definition of experiments and results visualization of an *in silico C. elegans* simulation. This GUI tool suite is available online to the scientific community (Si elegans Consortium, 2018c) and its source code has been made available at (Si elegans Web GUI tools Authors, 2018). The GUIs have been designed to be simulation tool-agnostic, thereby allowing for their integration with other virtual *C. elegans* simulation frameworks through the XML format experiment definition export (both behavioral experiment definition and neural network definition).

The graphical interaction concepts are grounded on concepts such as building block, drag-and-drop and virtual environment representation. The GUIs were developed using web technologies (Django web-framework, HTML5, CSS, AJAX, and JavaScript) to ensure ubiquitous public access to services and to warrant maximum compatibility with equipment by developing a solution that neuroscientists can use from standard web browsers. Furthermore, a common template system (Bootstrap, 2018) was used together with Web 3D technology to achieve consistent and intuitive web interfaces.

For the behavioral experiment definition, an input encoding strategy was introduced in the absence of an existing suitable encoding format in the literature. NeuroML describes the neuronal model design and connectome configuration. Joint neuronal activity and worm locomotion visualization were implemented. Each of the GUIs was presented in a series of case study examples (see Supplementary Videos 1–4).

Different experiment components (behavioral input, neuron models and neuron network configurations) have been defined using the GUI tools and shared with the public, and some emulations within *Si elegans* platform have been run, but for a large uptake and daily use by the scientific community, a fine-tuning of the complete *Si elegans* platform or a setup with software neural network simulator, sample models behaving similarly to real worm for different stimulus and validation procedures with *in vivo* experiments are required.

An acceptance study and an evaluation of the user experience of the GUI tools were carried out. The acceptance evaluation provided optimistic results with some improvement margin and the prioritization of add-on functionality briefly mentioned below.

In regard to the presented GUI approaches efficiency, in terms of computational resource usage the described GUI system would have comparable efficiency if implemented with simulation systems (e.g., pyLems or jLems) used in traditional computer simulation. *Si elegans* specific implementation is less optimal, since exclusive emulation is run due to a biological mimicking hypothesis behind this platform. In terms of saving researcher's time, it unites various interdependent tools that allow for a holistic workflow from neuron properties definition (down to the synaptic level) to visualizing the resulting behavior. It thus combines all required tools to visualize the behavioral outcome as a function of neuronal processes and interconnectivity, which would usually require the use of separate tools.

From a functionality perspective, future work will aim at refining and completing the behavioral experiment definition model though feedback from a wider scientific community in support of a richer behavioral repertoire. Stimuli application at neuronal level will be included as well. The GUI implementation of the behavioral experiment definition will be extended to allow users to define experiments with more natural interaction within the 3D window.

The Neuron Model Design GUI will support the extension of the neuron model library to include time-driven and event-driven models, with output via LEMS XML. Following community outreach results regarding future functionalities, non-electrical models inclusion will be studied together with the development of tools to optimize neuron model parameters and to test models during development.

While the Neural Network Configuration GUI permits the definition of *C. elegans*-specific networks, it is envisaged that an extensible graphical tool will be required to edit other networks. Together with a more advanced Neuron Model Design GUI, this would provide a complete solution for designing flexible neural network simulations through GUI tools alone. In line with this, user feedback has pointed to enabling selection of subnetworks for configuration. Currently, neuron variables for recording are automatically selected after network configuration. In the future, this selection will be made user-accessible.

Within the results visualization, in a similar fashion to the behavioral experiment definition GUI, it is planned to add more natural interaction within the 3D visualization window, visually representing the virtual experimenter behavioral interactions. Additionally, the functionality of enabling macro-analytics of neural trace data will be targeted. Finally, muscle activity will be recorded within the physics engine to be sent to the web for worm locomotion complimentary analysis.

## Author contributions

GE, FK, FC, and AM leaded the conceptualization and implementation of the tools and methods described in this paper. This work was carried under the supervision of FM and GE. AB looked after the biological coherence of the work. GE, AM, FC, PL, BM, RA, and FK worked on the implementation. All authors contributed, reviewed and approved the final version of the manuscript.

### Conflict of interest statement

The authors declare that the research was conducted in the absence of any commercial or financial relationships that could be construed as a potential conflict of interest.

## Supplementary material

Supplementary Table 1 Results of the Google form-based feedback questionnaires.

Supplementary Table 2 Results of the user experience measurement AttrakDiff tool.

Supplementary Video 1 Web-based behavioral experiment definition GUI demonstration video.

Supplementary Video 2 Neuron model definition GUI demonstration video.

Supplementary Video 3 Neuron network definition GUI demonstration video.

Supplementary Video 4 Results visualization GUI demonstration video.
