# Supplementary material for: Web-Based Interfaces for Virtual C. elegans Neuron Model Definition, Network Configuration, Behavioral Experiment Definition and Experiment Results Visualization
Source: Front Neuroinform. 2018 Nov 13;12:80. doi: 10.3389/fninf.2018.00080 (PMC6243129; doi:10.3389/fninf.2018.00080)
Supplement: Supplementary file 7 [file Data_Sheet_1.pdf]

Supplementary Material: Web-based Interfaces for Virtual *C. elegans* Neuron Model Definition, Network Configuration, Behavioural Experiment Definition and Experiment Results Visualisation

1 LOCOMOTION CALCULATION AND TRANSFER TO THE RESULT VISUALISATION GUI

Worm position and locomotion computation is too heavy to be executed as a web application. Thus, the PE is implemented in a dedicated server. The simulation in the PE uses a Finite Element Method (FEM) model and a tetrahedralised 3D representation of the worm with 95 muscles. These 95 muscles are located respecting the asymmetry of the real worm, in contrast to other locomotion modelling methods presented previously. Each individual muscle is defined by 32 tetrahedra. The rest of the body that fills the space between the muscles and the cuticle is composed of 7340 tetrahedra.

Locomotion is calculated by applying internal forces (elasticity and muscle activation) and external forces (gravity and friction) to the tetrahedra. Upon completion, results are transferred to the web for visualisation using a 3D model based on (Virtual Worm Project, 2018). To facilitate and speed up WebGL rendering, the number of polygons has been reduced from 1664120 in (Virtual Worm Project, 2018) to 354401 polygons (and from 831273 vertices to 277954). Besides providing the worm's outer shape, this 3D model also places the accurately shaped neurons in their correct position. The results visualisation GUIs (described in Results Visualisation UI section) allow the user to either show, highlight or hide the neurons while the worm locomotion is being rendered.

To synchronise the physics engine's locomotion output with the worm model web visualization,24 points are placed along the worm, and their position is transferred to the web interface and used to incrementally update the 3D animation of the worm. Once the positions of a 24 central point sample are computed and transferred to the web, 24 identical animation bones (Figure S1) are placed in the computed positions. These provide a simplified browser-based 3D worm model with identical shape of the one computed in the PE.

Equation S1 shows how the 24 central points are computed:

$$q_i = \frac{p_{vl,2i} + p_{dr,2i+1}}{2} \text{ for } i = 0, 1, \ldots, 23 \tag{S1}$$

where q_i is the position of the central point; $p_{vl,j}$ is the central point of the jth muscle in the ventral left row of muscles and $p_{dr,j}$ is the central point of the jth muscle in the dorsal right row of the worm model being used in the PE.

The information is transferred by means of a JSON file that contains the time of each timestep and the positions of the 24 central points in a row (72 float values, 3 float values to represent each XYZ position).

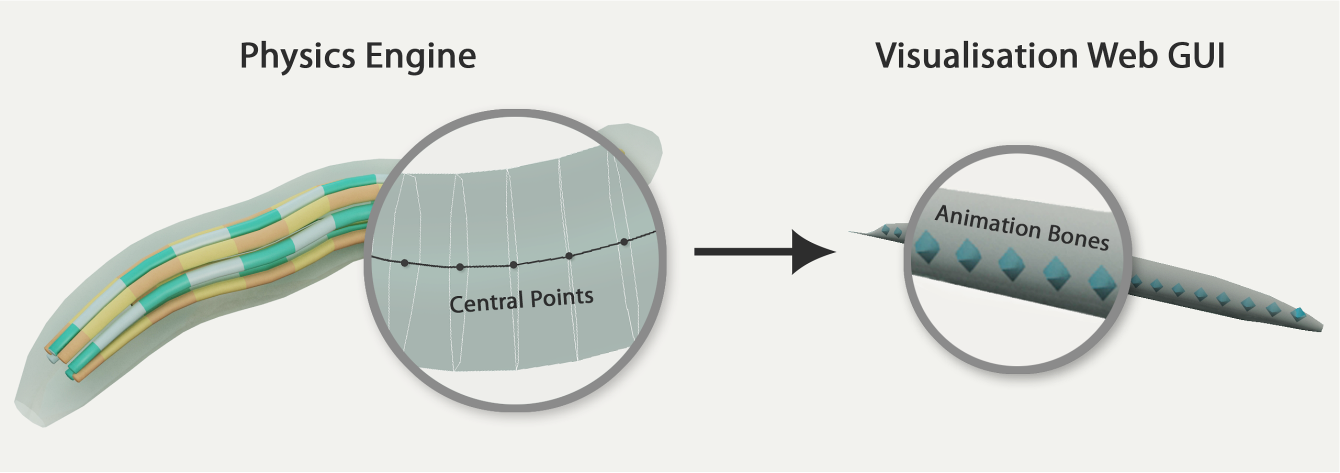

Figure S1: Worm position and pose transfer from PE to the visualisation web GUI. During simulation in the PE, a row of central points that encodes the shape of the worm at that moment is computed. This information is transferred to the visualisation web GUI and used to animate the 3D worm reproduction via animation bones technique

REFERENCES

Virtual Worm Project (2018). Wormbase - virtual worm home page. http://caltech.wormbase.org/virtualworm/ [last visit: 28 jul 2017]
