# Supplementary material for: Web-Based Interfaces for Virtual C. elegans Neuron Model Definition, Network Configuration, Behavioral Experiment Definition and Experiment Results Visualization
Source: Front Neuroinform. 2018 Nov 13;12:80. doi: 10.3389/fninf.2018.00080 (PMC6243129; doi:10.3389/fninf.2018.00080)
Supplement: Supplementary file 8 [file Data_Sheet_2.pdf]

Supplementary Material: Web-based Interfaces for Virtual *C. elegans* Neuron Model Definition, Network Configuration, Behavioural Experiment Definition and Experiment Results Visualisation

1 EXAMPLE USE CASES AND DETAILS OF THE USER INTERFACES

1.1 Example Behavioural Experiment Definition

The example depicted in Figure S1 shows two behavioural experiment inputs on the GUI experiment timeline, namely ”thermo”, and ”chemo”. The most detailed configuration shown corresponds to ”chemo”, for which the properties window is displayed on the right side. ”chemo” is of type *experiment-wide configuration*. In this experiment, the interaction is defined for the complete experiment duration. The chemotaxis type is set to ”Chemical Quadrants 2”. Quadrants and barrier chemicals are defined using a dropdown menu and their concentrations through slider widgets. ”thermo” is of *interaction from* $t0$ *to* $t1$ type.

The environment is configured by setting up the parameters including worm status, plate configuration, crowding and obstacles.

Figure S2 depicts the Behavioural Experiment Definition UI illustrating the worm status configuration block. For this example, the X and Y distances from the cylindrical plate are 0, and the angle with respect to the X axis is 0. No obstacles have been defined for this behavioural experiment. The supplementary material Video 1 demonstrates a web-based behavioural experiment definition GUI.

1.2 Example Neuron Model Definition

The screenshot in Figure S3 illustrates a basic ’integrate and fire’ neuron model. The numbered red box label has been included to identify the main areas of the user console screen, as follows:

1. The main navigation area is part of the main stylesheet template. This is a common template used by the Web-based GUI tools. It can be easily replaced if implemented in other installations.
2. This area contains buttons providing functionality for managing the model being displayed. ’Open’ enables users to select from models which they have previously saved, or those shared by other users. ’Show XML’ allows users to view the LEMS XML for the model.
3. The Model Details Panel presents the details of the currently selected component. The presented selected component is of type ’iafRefCell’, an integrate-and-fire cell with capacitance. The parameters of each component can be entered by the user. The Model Details Panel includes a button for any building blocks which can be linked to. The presented component links only with synapses (displayed in a selection box) that inherit from type ’basePointCurrent’. In this case (Figure S4), synapses of type ’expTwoSynapse’ ’expOneSynapse’ have been added.

4. The Model Definition Workspace panel enables drag and drop placement of model components to the current model. Clicking on a component causes its details to be shown and edited in the Model Details Panel.
5. The Component Selection Panel allows users to browse and select from currently available model components. The selected model component details (name, description, dynamic behaviour etc.) are shown in the left-hand-side model details panel. This panel is also used to select components from the library or from user-customised components. In this panel, the user can browse to the required component, and drag (add) the component box into the Model Definition Workspace panel. Components may be tagged in the database with free form tags, to facilitate component browsing. The first component to be added to the model always inherit from the building block 'baseCell', since all neuron models on the developed system should represent a neural cell.

1.3 Example Neural Network Configuration

In this case, a network is being defined, with some of the AS (ventral cord motor) neurons currently selected. The numbered red box labels have been drawn onto the screenshot to identify the main areas of this screen. The use of each of these areas is as follows:

1. The main navigation area as previously described.
2. Buttons providing functionality for managing the network currently being displayed.
3. 3D Network View: This Panel shows a 3D representation of all neurons in *C. elegans*. As neurons are selected by the user, they are highlighted and all other neurons are faded. This allows the user to browse for specific neurons and to see how neurons relate to each other in physical space. Note that the neural network being configured is composed of point neurons, and the morphology is only displayed to illustrate neuron position and connectivity. The 3D viewer is based on the Worm Browser which is part of the OpenWorm project (OpenWorm, 2018). Navigation is possible with the mouse (drag and scroll) and with the keyboard arrow buttons.
4. Network Details Panel: This panel is the main working area for specifying the details of the network; the displayed data changes depending on the current activity of the user. The three main display modes (Neuron Selection Mode, Neuron Parameter Mode, and Synapse Selection Mode) are described in the following sections.

1.3.1 Neuron Selection Mode

The Neuron Selection Mode is the default mode, displayed in Figure S5. In this mode, the following components are shown:

- The top dropdown menu shows the neuron model to be used for any neuron not customised by the user.
- The select box on the left lists all *C. elegans* neurons. Clicking on a neuron (or selecting multiple neurons) highlights the 3D view. Double-clicking on a neuron opens its information page on WormAtlas (Altun and Hall, 2002).
- The select box on the right displays a list of all neurons, or groups of neurons, which have been customised in this network. This allows users to browse or edit customisations.
- The customise button is used when a user wishes to change some of the details for the currently highlighted neuron(s). When this button is clicked, the Network Details Panel changes to Neuron Parameter Mode. If more than one neuron is selected, the user is prompted to enter a name for this group of neurons. This name is shown in the "customised" select box on the right-hand side of the

panel. Note that users may customise as many or as few neurons as they wish. If no neurons are customised, then the network uses the default model with all of the parameters as defined in the Model Definition GUI. All synaptic connections use the default synapse type.

1.3.2 Neuron Parameter Mode

This mode is available to the user to specify the parameters for the neuron(s) that have been selected for customisation. The screenshot in Figure S6 shows an example of Neuron Parameter Mode. In this case, the neurons AS1-AS11 have been selected for customisation. 'AS' is the group name given by the user when the 'Customise' button was pressed. The list of neurons in the group is shown in Figure S6.

The Model dropdown allows the user to specify the neuron model (as defined in the Neuron Model Design GUI) to use for this neuron or set of neurons.

The Component dropdown allows the user to move between the building blocks in the model, setting values for the parameters in each building block. Each time the dropdown selection changes, the parameter input boxes below it also changes.

The 'Synapses' button moves the Network Details Panel into Synapse Selection Mode.

The 'Uncustomise' button removes all customisations for this neuron or group of neurons and effectively removes it from the customised list.

The 'Done' button returns the Network Details Panel to Neuron Selection Mode, saving all customisations.

Supplementary material Video 3 demonstrates the Neuron Network Definition GUI usage.

1.3.3 Synapse Selection Mode

This mode is used by a user to specify the synapse type for the customised neuron(s). The screenshot in Figure S7 shows an example of the Synapse Selection Mode. In this case, only the neuron AS1 was selected for customisation.

The 'Default Synapse' dropdown allows the user to specify which synapse type to use for any synaptic connection that does not have an explicit synapse type set.

Note that customisation of individual synapses is only available when customising a single neuron. When customising groups of neurons, only the default synapse type can be changed. This is because different neurons have different numbers of synapses. The entries in the table allow the user to specify a specific synapse type for individual synaptic connections. The 'Number' field in Figure S7 refers to the number of synapses between the 2 neurons in question. This field is populated by the standard *C. elegans* connectome specified in WormAtlas.

1.4 Example Results Visualisation

Figure S8 and Figure S9 illustrate the visualisation of synthetic data generated by the PE (Mujika et al., 2014) for locomotion, and the associated action potentials. It depicts the worm's position and shape at second 3.3 in an experiment that lasted 23 seconds. The timings can be controlled either on the timeline using the Play / Pause button or at the X axis of the neural trace and behavioural stimuli mode visualisation graphs.

Figure S8 illustrates the behavioural stimuli composed of an experiment-wide stimulus type named "thermo", an interaction from $t0$ to $t1$ type stimulus named "galvano" and an interaction at specific time t named "touch".

Figure S9 presents the action potential plot for the experiment. ADAL and PDEL neurons are shown. The scroll facility is activated whenever the number of selected element traces does not fit into the visual timeline window. The scroll enables navigation of the selected neuron traces.

REFERENCES

Altun, Z. and Hall, D. (2002). Wormatlas. http://www.wormatlas.org [[last visit: 31 jan 2018]

Mujika, A., Epelde, G., De Mauro, A., and Oyarzun, D. (2014). Visualization of a virtual caenorhabditis elegans in webgl. In *Neurotechnix: 2nd InternationalCongresson Neurotechnology, Electronicsand Informatics* (SCITEPRESS - Science and Technology Publications), 164–168

OpenWorm (2018). Openworm bro2018. http://browser.openworm.org/ [last visit: 31 jan 2018]

FIGURES

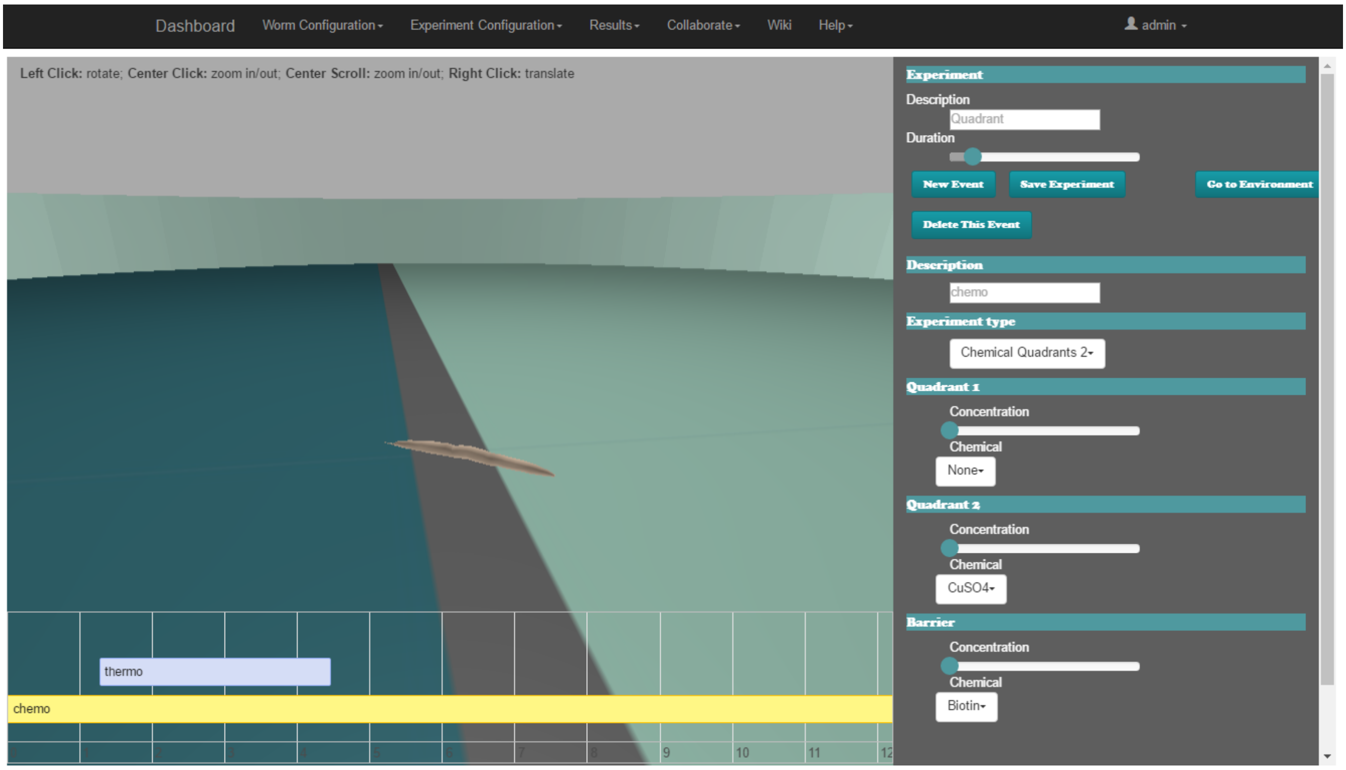

Figure S1: Web-based C. elegans behavioural experiment definition GUI for configuring behavioural input parameters. 3D window (middle), experiment definition window (bottom) and the properties window (right).

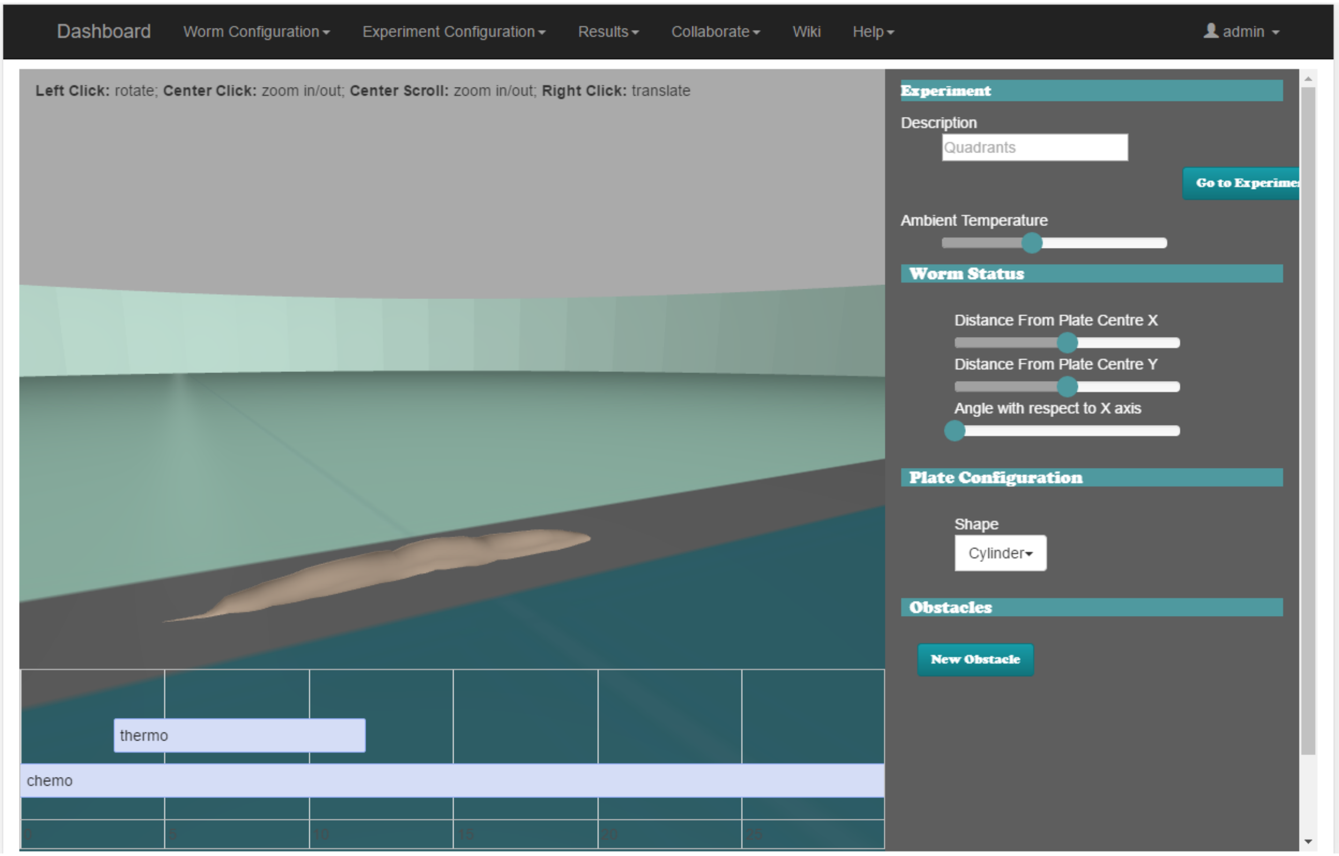

Figure S2: Web-based behavioural experiment definition GUI for configuring the environment parameters.

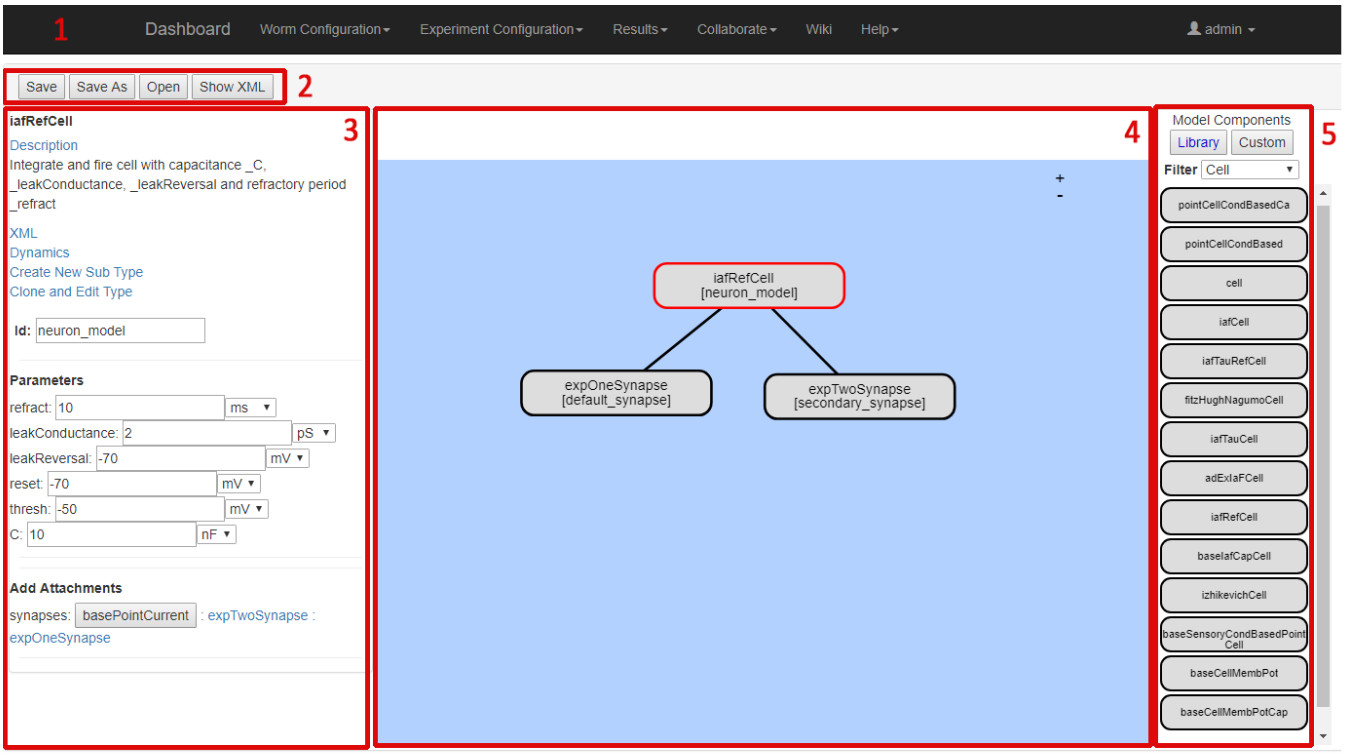

Figure S3: Neuron Model Definition Screen.

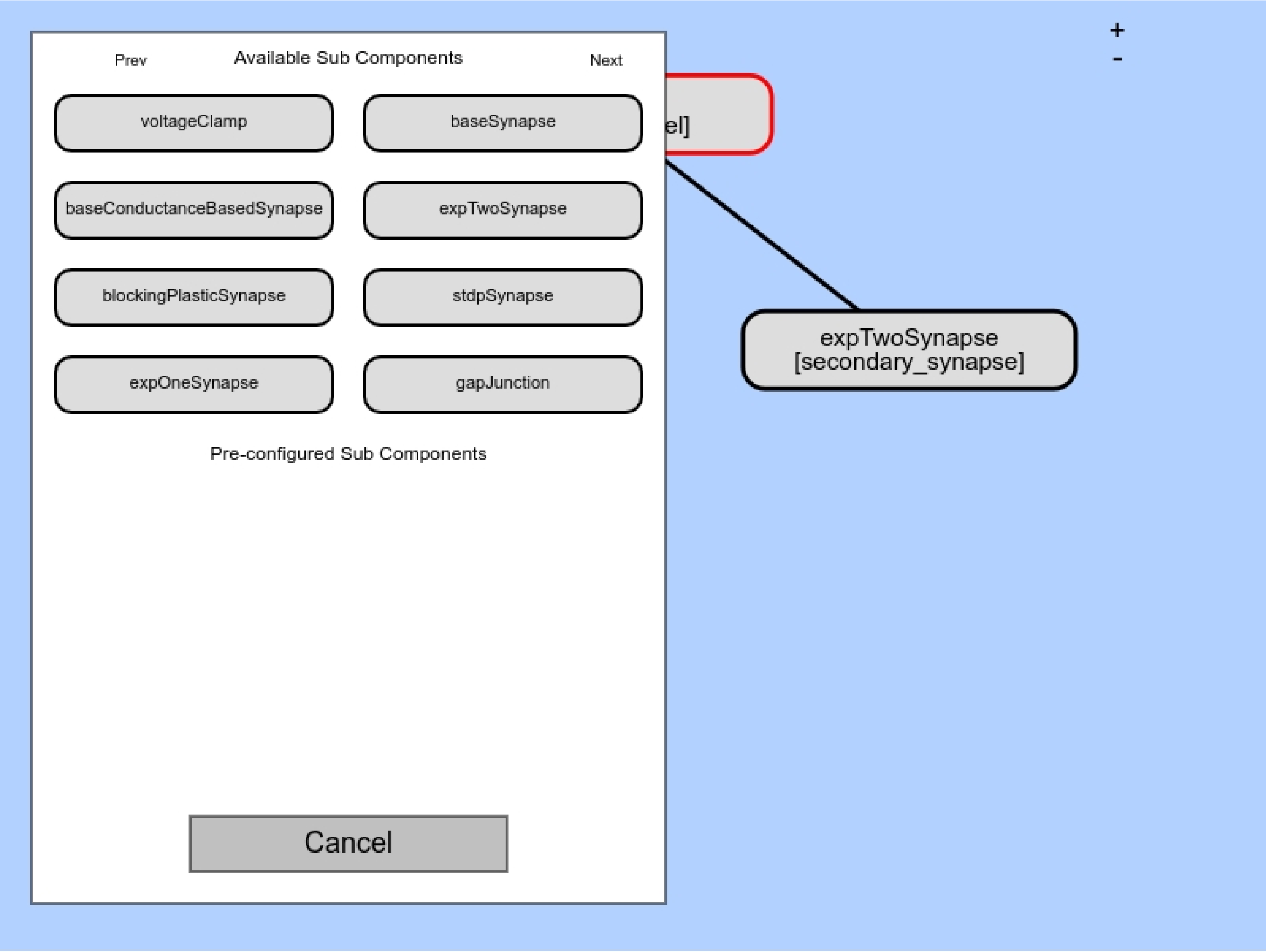

Figure S4: GUI screen for selection of Synapse Building Block.

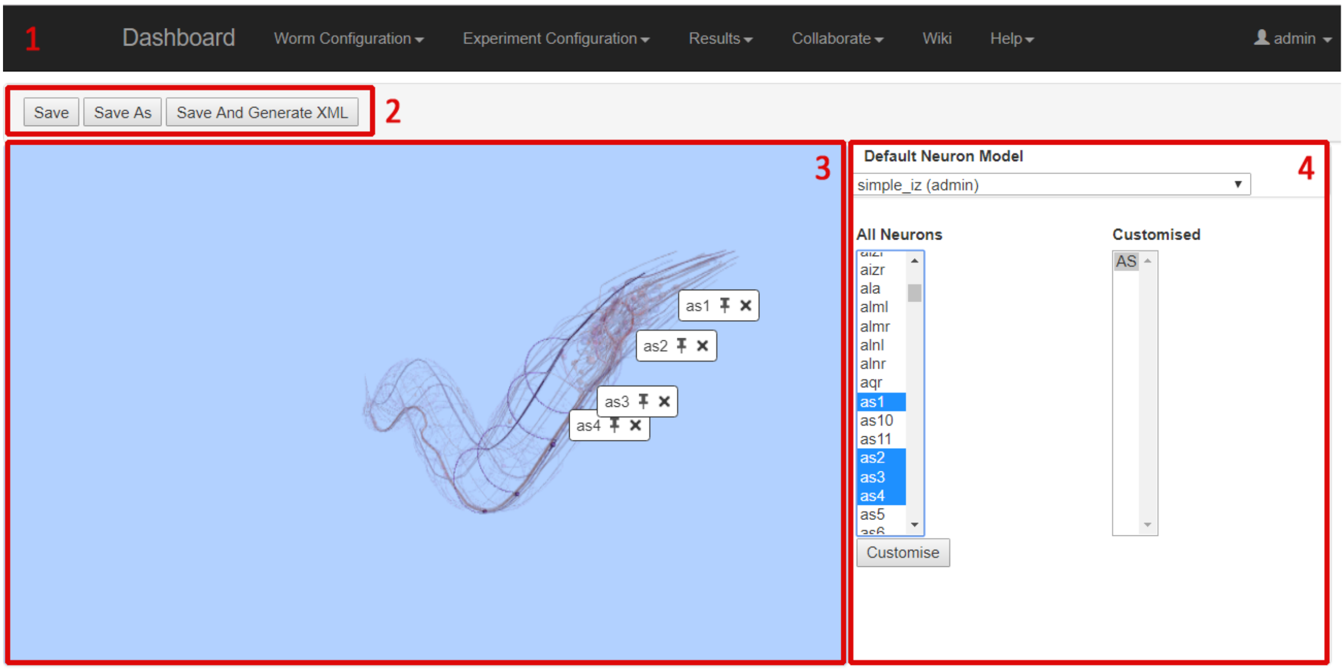

Figure S5: Neural Network Configuration GUI.

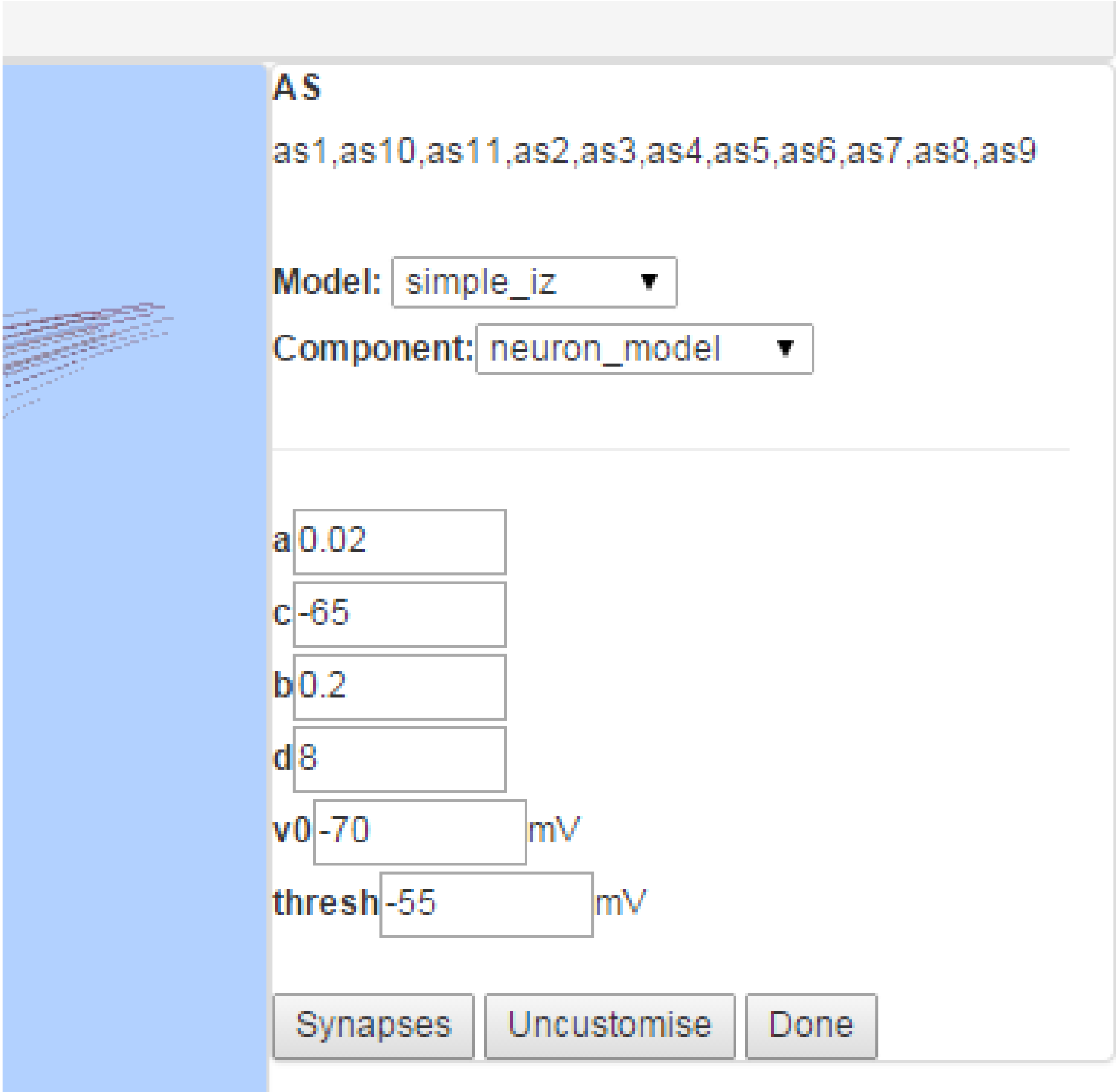

Figure S6: Network Details Panel in Neuron Parameter Mode.

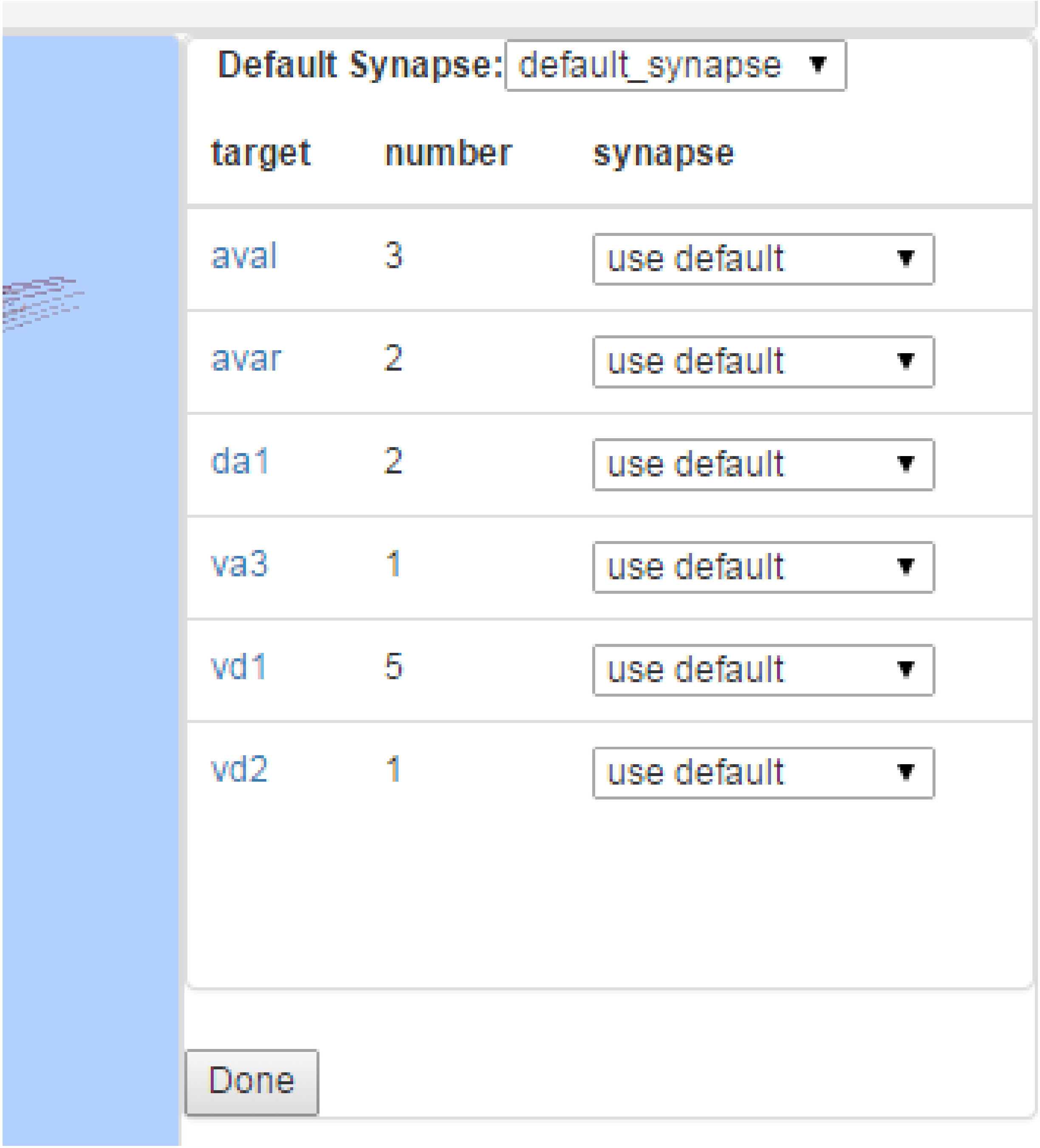

Figure S7: Network Details Panel in Synapse Selection Mode.

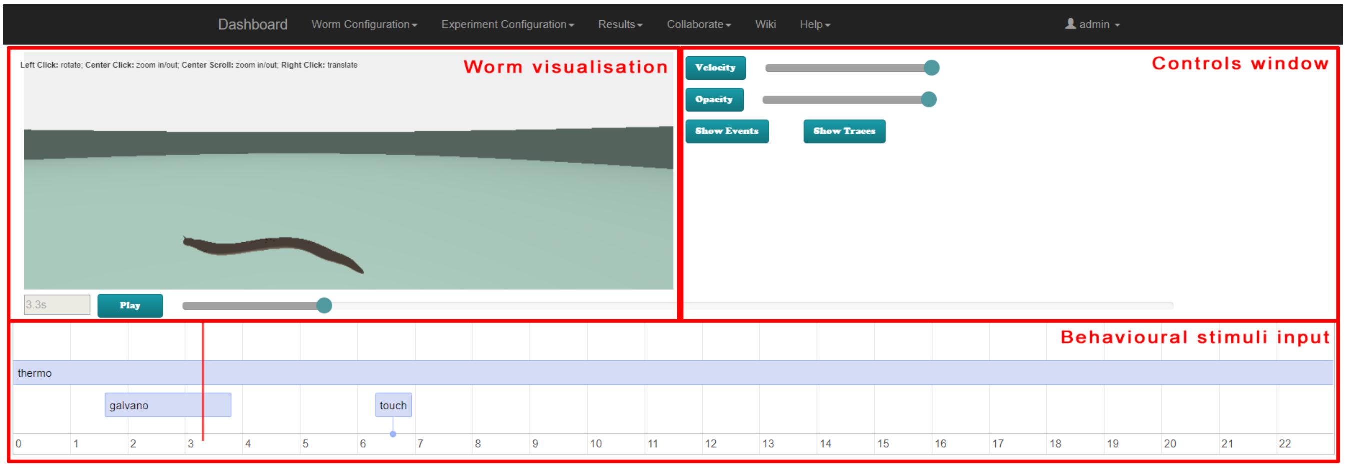

Figure S8: Web-based virtual worm simulation results visualisation with behavioural stimuli input. The virtual worm visualisation is synchronised with behavioural stimuli at the bottom and can be controlled by sliders on the right-hand side and Play / Pause button.

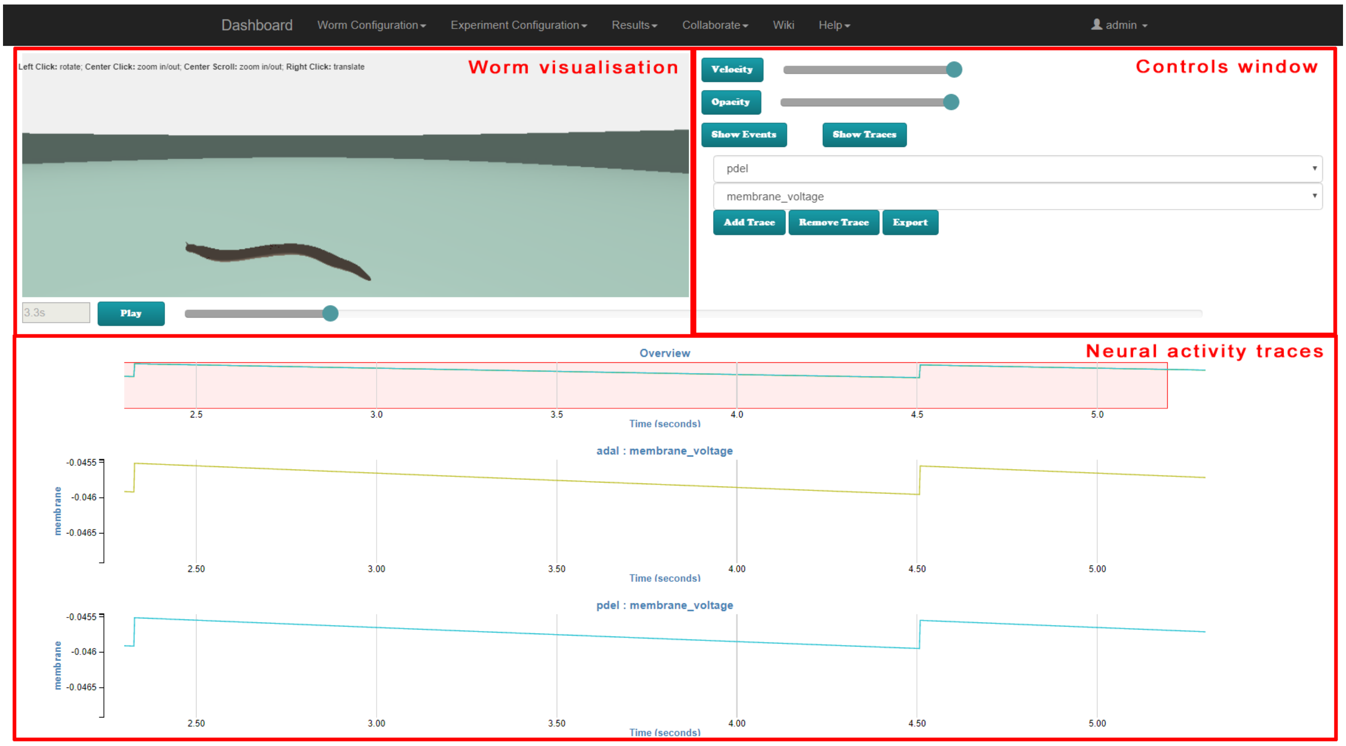

Figure S9: Web-based virtual worm simulation results visualisation with neural traces visualisation. The virtual worm visualisation is synchronised with the neural activity at the bottom. Parameters can be selected in controls window on the right-hand side, and can be controlled by using the slider on the right-hand side and Play / Pause button.
