# Supplementary material for: Web-Based Interfaces for Virtual C. elegans Neuron Model Definition, Network Configuration, Behavioral Experiment Definition and Experiment Results Visualization
Source: Front Neuroinform. 2018 Nov 13;12:80. doi: 10.3389/fninf.2018.00080 (PMC6243129; doi:10.3389/fninf.2018.00080)
Supplement: Supplementary file 9 [file Data_Sheet_3.PDF]

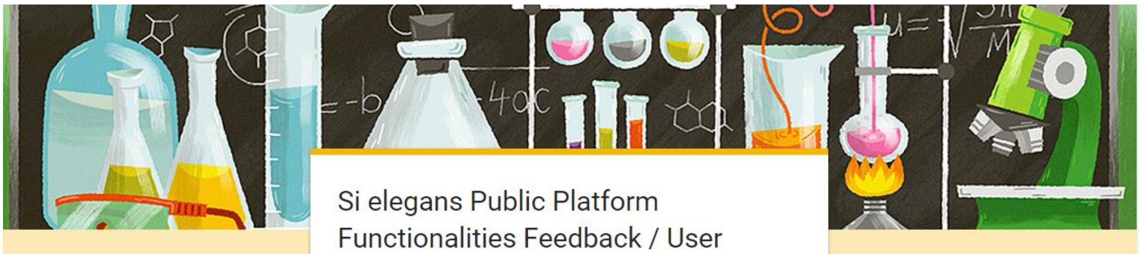

Si elegans Public Platform Functionalities Feedback / User Experience Assessment

We would love to hear your thoughts or feedback on how we can improve your experience!

* Required

Responder details

Name

Your answer

Email

Your answer

Would you be willing to be contacted for further functionality discussion? *

- ◯ Yes
- ◯ No

NEXT

Page 1 of 12

Never submit passwords through Google Forms.

Neuron Model Design

Please provide us with specific feedback on neuron model design tools

https://platform.si-elegans.eu/booking/view_NeuronModels

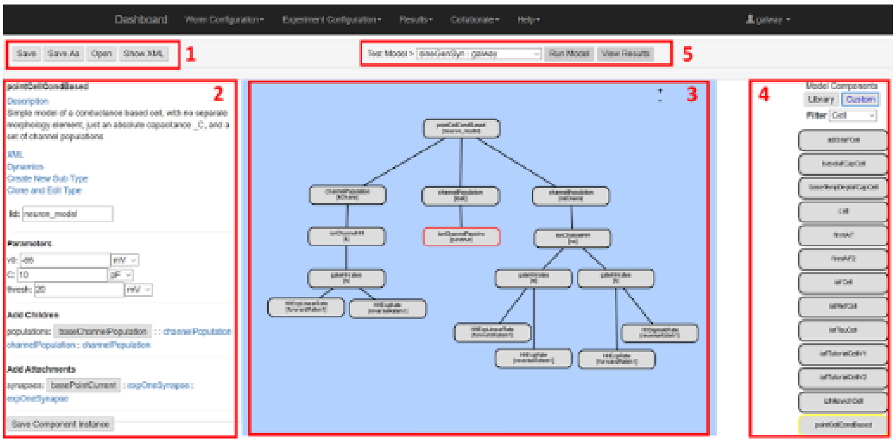

Functionality Feedback

Your answer

Evaluate Possible Future Functionalities

Improve the testing methods for neurons under development

	1	2	3	4	5	
Not Important	○	○	○	○	○	Very Important

Expand library of neuron models to include non-electrical models (not ion-based or voltage-based) abstract models

	1	2	3	4	5	
Not Important	○	○	○	○	○	Very Important

Suggestions for Other Functionalities

Your answer

Neuron Network Configuration

Please provide us with specific feedback on neuron network configuration tool

https://platform.si-elegans.eu/booking/view_NeuralNetworks

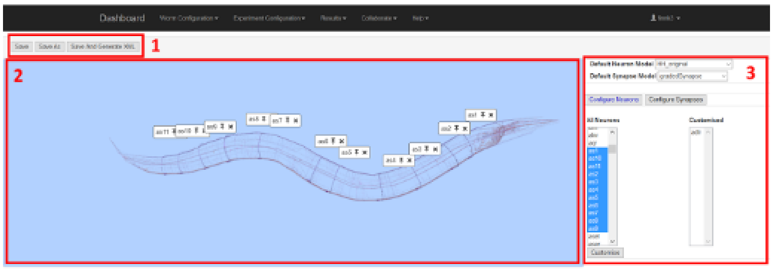

Functionality Feedback

Your answer

Evaluate Possible Future Functionalities

Enable selection of subnetworks for configuration

	1	2	3	4	5	
Not Important	○	○	○	○	○	Very Important

Allow for the running of a network without a physics engine (standalone simulations)

	1	2	3	4	5	
Not Important	○	○	○	○	○	Very Important

Suggestions for Other Functionalities

Your answer

Behavioural Experiment Design

Please provide us with specific feedback on behavioural experiment design tool

https://platform.si-elegans.eu/behaviouralExperimentDefinition/experimentDefinition_Selection

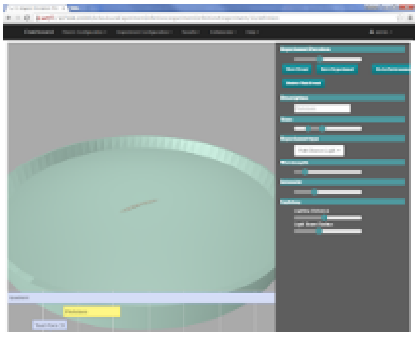

Functionality Feedback

Your answer

Evaluate Possible Future Functionalities

Improve Experiment Definition 3D Realism - e.g. specify interaction are by clicking on the worm

	1	2	3	4	5	
Not Important	○	○	○	○	○	Very Important

Richer Behavioural Repertoire

	1	2	3	4	5	
Not Important	○	○	○	○	○	Very Important

Allow for Direct Stimuli Specification to Neuron

	1	2	3	4	5	
Not Important	○	○	○	○	○	Very Important

Suggestions for Other Functionalities

Your answer

Locomotion Results Visualization

Please provide us with specific feedback on locomotion results visualization

https://platform.si-elegans.eu/booking/view_PE_results

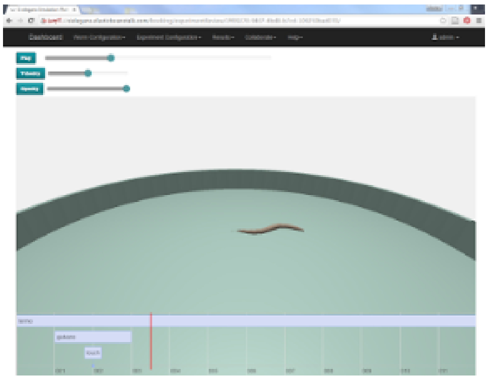

Functionality Feedback

Your answer

Evaluate Possible Future Functionalities

Represent Virtual Experimenter Behavioural Interactions - 3D Realism

	1	2	3	4	5	
Not Important	◯	◯	◯	◯	◯	Very Important

Visualise Muscles Contraction and Relaxation

	1	2	3	4	5	
Not Important	◯	◯	◯	◯	◯	Very Important

Suggestions for Other Functionalities

Your answer

Neuron Results Visualization

Please provide us with specific feedback on Neuron Results Visualization

https://platform.si-elegans.eu/booking/view_RB_results

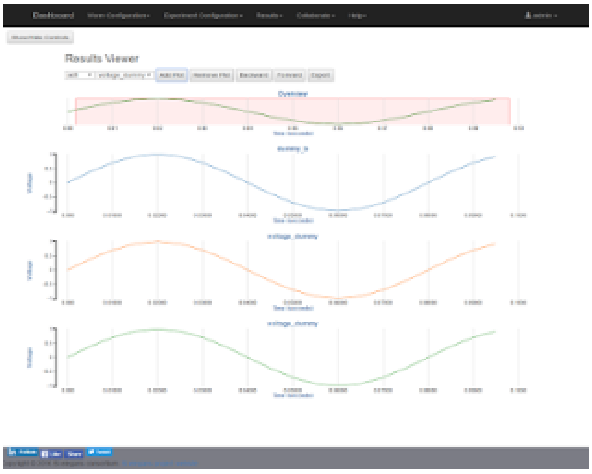

Functionality Feedback

Your answer

Evaluate Possible Future Functionalities

Enable export to HDF5 format

	1	2	3	4	5	
Not Important	○	○	○	○	○	Very Important

Enable macro analytics (activity graphs and PCA)

	1	2	3	4	5	
Not Important	○	○	○	○	○	Very Important

Suggestions for Other Functionalities

Your answer

Integrated Results Visualization

Please provide us with specific feedback on the Integrated Results Visualization

https://platform.si-elegans.eu/booking/view_integrated_results

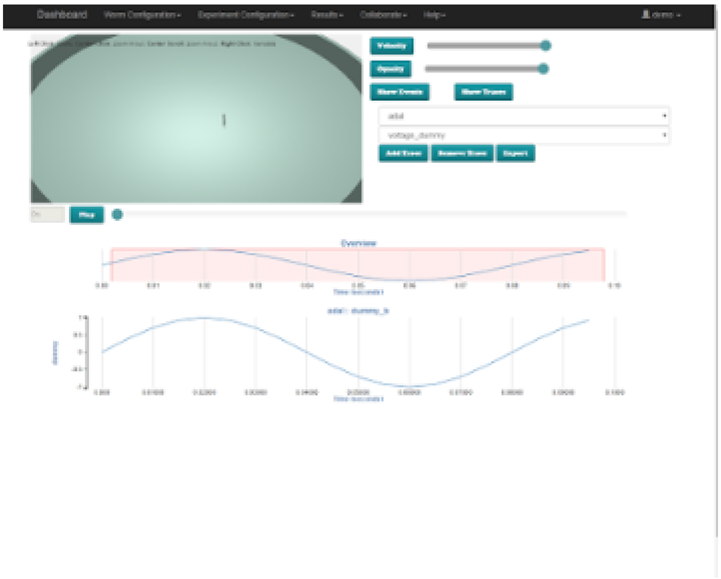

Functionality Feedback

Your answer

Evaluate Possible Future Functionalities

Enable export to HDF5 format

	1	2	3	4	5	
Not Important	◯	◯	◯	◯	◯	Very Important

Enable macro analytics (activity graphs and PCA)

	1	2	3	4	5	
Not Important	◯	◯	◯	◯	◯	Very Important

Suggestions for Other Functionalities

Your answer

General Usability - Usefullness Feedback

Please provides us some feedback from a general perspective

Do you prioritise execution time of your tools over usability on your daily use tools?

	1	2	3	4	5	
Strongly Disagree	○	○	○	○	○	Strongly Agree

Please provide us with a general feedback on our GUIs

Your answer

Do you require the ability to simulate sub networks of the connectome?

- ○ Yes
- ○ No

Would you make experiment in a in-silico emulation platform like Si elegans

- ○ Yes
- ○ No

Would you trust to do your experiments on an online platform or would you rather verify them locally?

	1	2	3	4	5	
Locally only	○	○	○	○	○	Online only

If yes,... Please specify which usage you would give to this platform

Your answer

If not, ...Please tell us what you're missing. (e.g Which general functionality you're missing.)

Your answer
