# Supplementary material for: Web-Based Interfaces for Virtual C. elegans Neuron Model Definition, Network Configuration, Behavioral Experiment Definition and Experiment Results Visualization
Source: Front Neuroinform. 2018 Nov 13;12:80. doi: 10.3389/fninf.2018.00080 (PMC6243129; doi:10.3389/fninf.2018.00080)
Supplement: Supplementary file 10 [file Data_Sheet_4.pdf]

Supplementary Material:
Web-based Interfaces for Virtual *C. elegans* Neuron Model Definition, Network Configuration, Behavioural Experiment Definition and Experiment Results Visualisation

1 ACCEPTANCE EVALUATION DESCRIPTIVE ANALYSIS

1.1 Future functionality prioritisation

For the behavioural experiment definition GUI tool, already suggested functionalities such as an increase in experiment definition realism by specifying the stimulus interaction area through a click on the worm was valued neutrally ($\bar{x} = 3.08$ out of 5, $s = 0.99$). In contrast, having a richer behavioural repertoire was considered rather important ($\bar{x} = 3.25$, $s = 1.05$), and allowing for stimuli application at neuronal level as most important ($\bar{x} = 3.75$, $s = 1.21$). In the free entry text submissions, the option of running simulations for colonies of worms was proposed.

In regards to the neuron model design GUI suggested functionalities, ”improve the testing methods for neurons under development” ($\bar{x} = 3.83$, $s = 0.93$) and ”expand library of neuron models to include non-electrical models (not ion-based or voltage-based) abstract models” ($\bar{x} = 3.25$, $s = 1.05$) were graded as rather important but not critical. User-suggested functionalities include tools to optimise neuron model parameters, inclusion of non-spiking neuron models, and support to integrate models pre-designed in other programming languages. Users also suggested some visual improvement for enhancing viewing of dependencies, function and completeness of a model.

Within the suggested functionalities for the neural network configuration GUI tool, the selection of subnetworks for configuration was identified as very important ($\bar{x} = 4$, $s = 0.85$), while running standalone neural simulations (without a physics engine involved) was rated as rather important ($\bar{x} = 3.41$, $s = 1.44$). Users suggested an option to display the configured network (synapses).

Within the suggested functionalities for results visualization GUI, the neural trace data export to HDF5 was valued as neutral ($\bar{x} = 3$, $s = 0.89$), while enabling macro-analytics of neural trace data was valued as rather important ($\bar{x} = 3.42$, $s = 0.97$). Regarding the locomotion window, representing virtual experimenter behavioural interactions with 3D realism was valued as rather important ($\bar{x} = 3.6$, $s = 0.98$) as was visualising muscles contraction and relaxation during locomotion ($\bar{x} = 3.41$, $s = 1.24$). Users proposed the simultaneous visualisation of stimuli, neurons and locomotion.

1.2 Descriptive analysis of the user experience evaluation results

Figure S1 includes a list of all word-pair ratings in AttrakDiff with mean values per each word-pair for the tested GUI tools. Word-pairs rated with extreme values indicate characteristics which are either critical or well-resolved. Users tended to rate the GUI tools as good, innovative and practical. An outlier or critical value is identified in the cautious-bold word pair, which contrasts with the inventive and innovative rating,

but can be understood in the scope of a comment made by a user, stating that the user found the interface intuitive and cautiously designed. Users defined the integrated web GUIs as clearly structured within the same web style-sheets and well-ordered according to their place in the logical execution of the emulation.

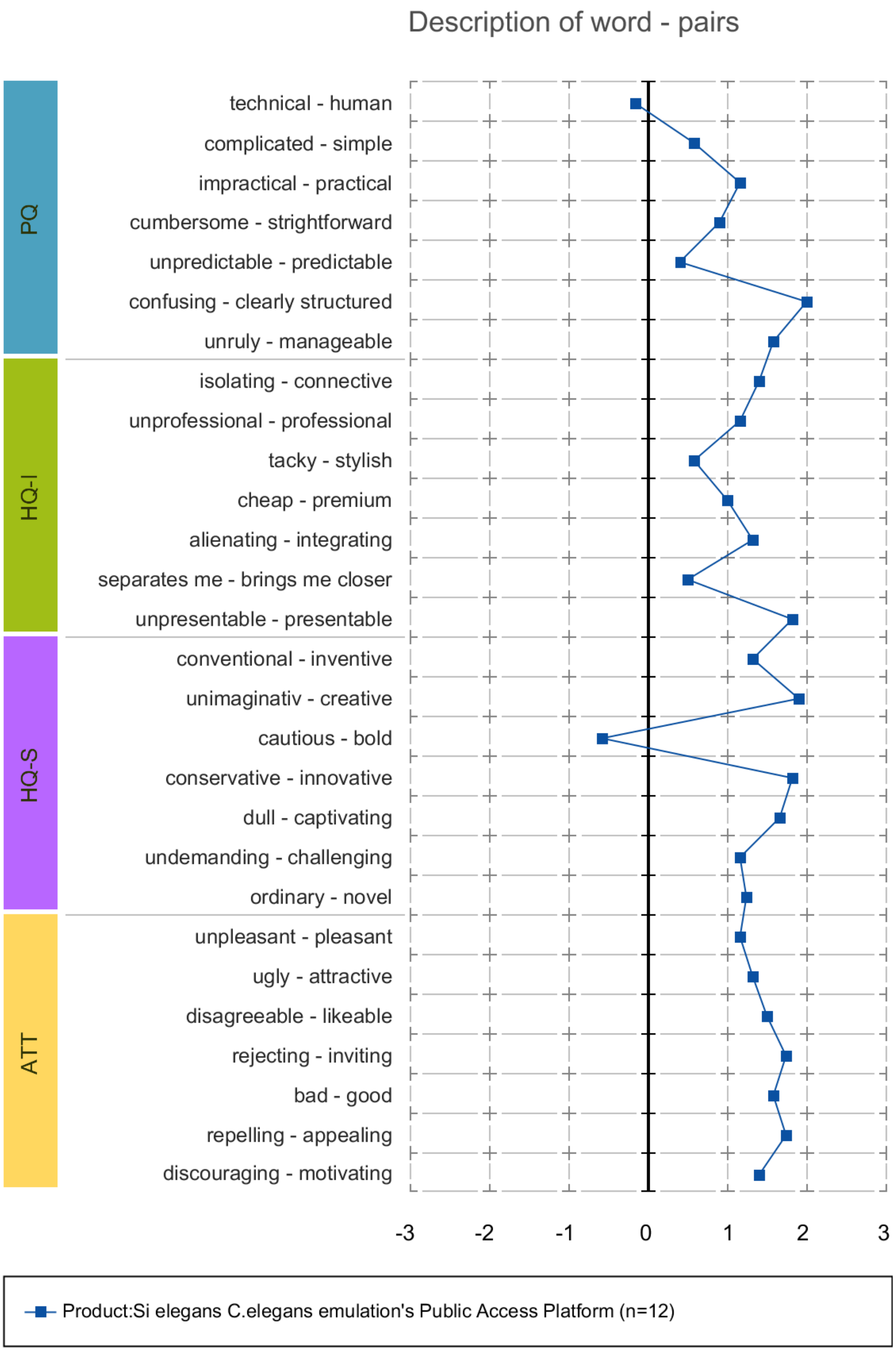

Figure S1: Mean ratings of word pairs

Figure S2 shows the characteristics of the questionnaire items by averaging them on the level of the AttrakDiff dimensions. All mean values for the dimensions lie above the mean value of the rating scale. The best value has been achieved in the attractiveness dimension (ATT). This value reflects the indications

which users previously made regarding the ease-of-use and visual nature of the platform. The lowest mean value was achieved in the pragmatic quality dimension (PQ). This can be understood considering that the proposed GUI tools concept is new and it will take time for dissemination and acceptance consideration. According to the AttrakDiff results, the tested application is within the standards in this discipline. At the same time, the application has improvement margin as shown by the mean value scoring of the AttrakDiff dimensions.

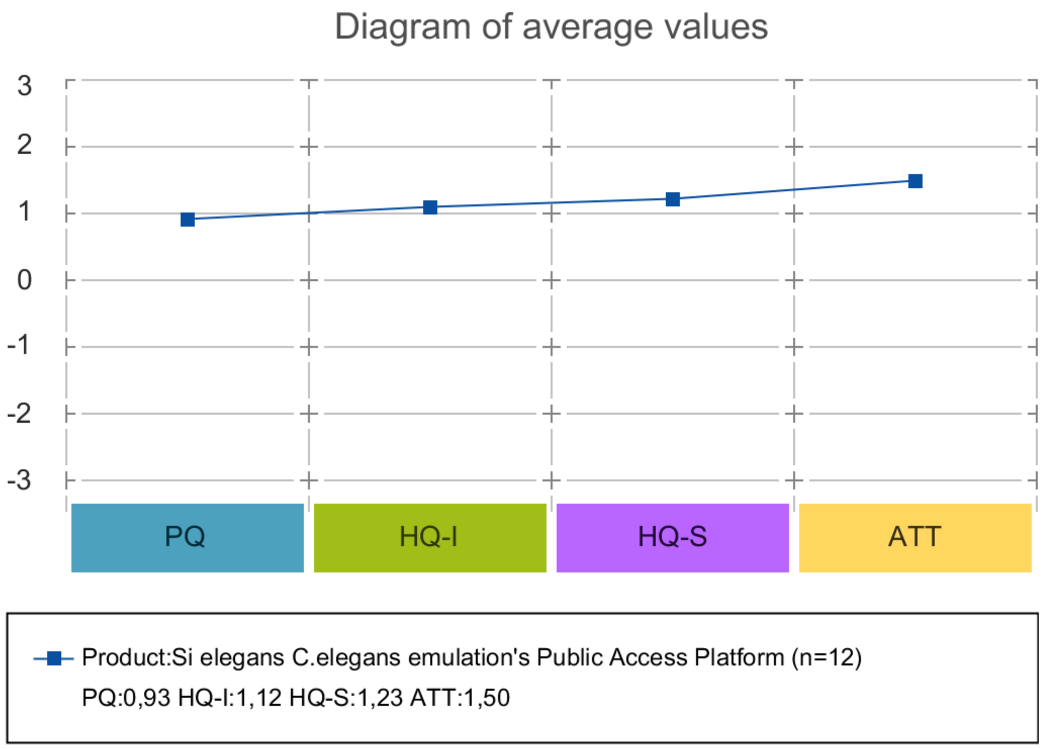

Figure S2: Mean value of all four AttrakDiff dimensions

Pragmatic quality (usefulness and usability of the system) and hedonic quality (motivation, stimulation and challenge for the user) are independent and both influence attractiveness. AttrakDiff provides an overview of the tested application on these dimensions and classifies the application into a character region (Figure S3). The assessment of the tested application is close to the top-right desired area, but still in the frontiers of task-oriented (good as mean for achieving the goal, but regular on user stimulation) and self-oriented (good for user stimulation, but regular for the achieving of system's goals), and with improvement margin as the confidence interval shows.

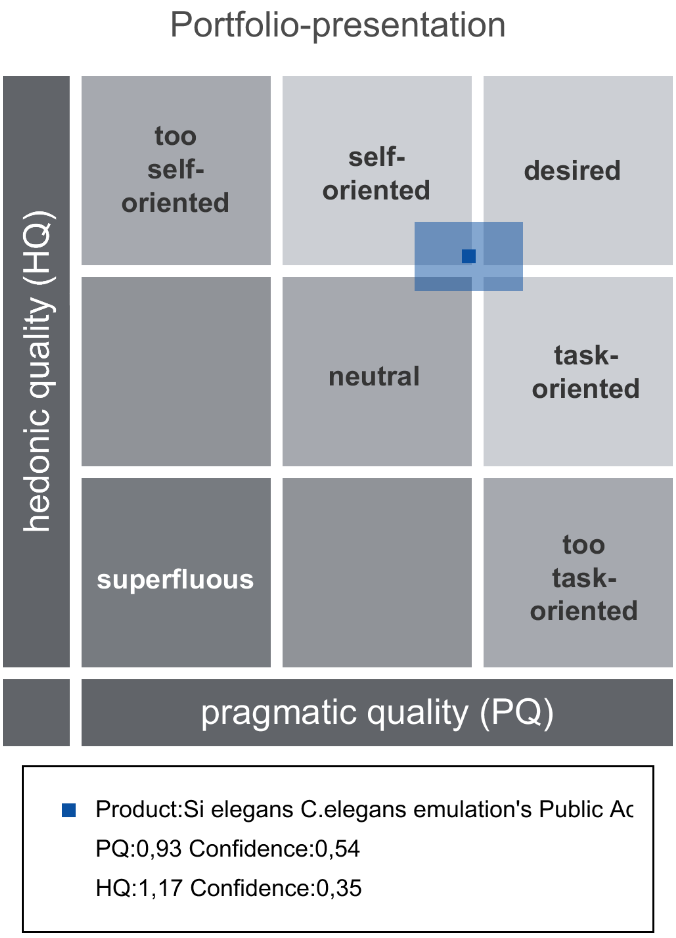

Figure S3: Results overview presenting PQ and HQ dimensions and the confidence rectangle
